# Four hub genes regulate tumor infiltration by immune cells, antitumor immunity in the tumor microenvironment, and survival outcomes in lung squamous cell carcinoma patients

**DOI:** 10.18632/aging.202351

**Published:** 2021-01-10

**Authors:** Tuo Zhang, Haitang Yang, Beibei Sun, Feng Yao

**Affiliations:** 1 Department of Thoracic Surgery, Shanghai Chest Hospital, Shanghai Jiao Tong University, Shanghai, China; 2 Institute for Thoracic Oncology, Shanghai Chest Hospital, Shanghai Jiao Tong University, Shanghai, China

**Keywords:** immunity, lung squamous carcinoma, single sample gene set enrichment analysis, robust rank aggregations, CIBERSORT

## Abstract

In this study, we performed bioinformatics analyses to identify hub genes that regulate tumor infiltration by immune cells and antitumor immunity in the lung squamous cell carcinoma (LUSC). We identified 1738 robust and stable differentially expressed genes (DEGs) in the LUSC tissues based on robust rank aggregation (RRA) analysis of RNA-sequencing data from 5 GEO-LUSC datasets. We then classified TCGA-LUSC patients based on ssGSEA and ESTIMATE analyses of LUSC tissues into high, medium and low immunity subgroups showing significant differences in tumor purity. Weighted gene co-expression network analysis of the robust DEGs revealed five immunity-related modules, including the brown module with 762 DEGs and 30 hub genes showing the highest correlation with the immunity-related LUSC patient subgroups and their clinicopathological characteristics. We selected four hub genes, *LAPTM5, C1QC, CSF1R* and *SLCO2B1*, for validation of the immunity status and prognosis of LUSC patients. High expression of these four genes correlated with increased infiltration of immune cell types, upregulation of the immunosuppressive TOX pathway genes, CD8^+^ T cell exhaustion, and shorter overall survival of LUSC patients. These findings demonstrate that four hub genes regulate tumor infiltration of immune cells, anti-tumor immunity, and survival outcomes in LUSC patients.

## INTRODUCTION

Lung cancer is the most common cancer worldwide that originates from the bronchial mucosal glands and is associated with poor prognosis because of late diagnosis [1–3]. Lung squamous cell carcinoma (LUSC) is the second most common histological type of lung cancer that originates from the main bronchus, develops into a sessile mass, and blocks the lumen. In recent years, targeted therapy has improved the survival and quality of life of nearly 60% of lung adenocarcinoma patients that carry the corresponding driver gene mutations [4, 5]. However, majority of the advanced lung squamous cell carcinoma (LUSC) patients do not harbor mutations in the driver genes that are amenable for the currently available targeted therapeutics and are therefore treated by traditional chemotherapies [4]. In recent years, immune check point block (ICB) therapeutics have been available for treating LUSC patients [6], but, some LUSC patients show hyper-progression after ICB treatment [7]. Several cytokines including IL-6 promote the stemness of the LUSC cells in the tumor microenvironment and assist the tumor cells to escape immune surveillance [8, 9]. Evasion of immune surveillance and chronic inflammation are both hallmarks of tumor progression in all tumor tissues including LUSC [10]. In the past decade, RNA-sequencing technology has developed greatly and helped to gain greater insights into the molecular mechanisms that are promote or restrict growth and progression of LUSC [11]. However, majority of these studies have small sample sizes. Moreover, the use of different microarray platforms reduces the statistical power while merging data from different cohorts [5].

In this study, we performed Robust Rank Aggregation (RRA) analysis of 5 microarray datasets from the Gene Expression Omnibus (GEO; http://www.ncbi.nlm.nih.gov/geo/) database to identify differentially expressed genes (DEGs) between LUSC tissues and matched normal lung tissue samples [12]. We then quantified the enrichment levels of 29 immune signatures in each LUSC sample from the TCGA database using gene set enrichment analysis or ssGSEA [13, 14] and identified different immunity-related subtypes of LUSC patients. We also used the ESTIMATE algorithm [15] to evaluate the immune cell infiltration levels (immune score), tumor purity, and stromal content (stromal score) in all LUSC samples, and performed weighted gene co-expression network analysis (WGCNA) of the DEGs to identify key immunity-related modules and annotated the functions of the DEGs in the highest correlating module using Gene Ontology (GO) and Kyoto Encyclopedia of Genes and Genomes (KEGG) analyses. We selected four hub genes, *LAPTM5*, *C1QC*, *CSF1R*, and *SLCO2B1*, and analyzed their correlation with the tumor infiltration of 22 immune cell subsets using CIBERSORT [16] and the prognosis of different immunity-related LUSC patient subtypes. Finally, we performed Gene Set Enrichment Analysis (GSEA), and Gene Set Variation Analysis (GSVA) to determine the potential functions of these four hub genes.

## RESULTS

### Identification of robust DEGs by the RRA method and functional enrichment analysis of DEGs

**Figure 1:**
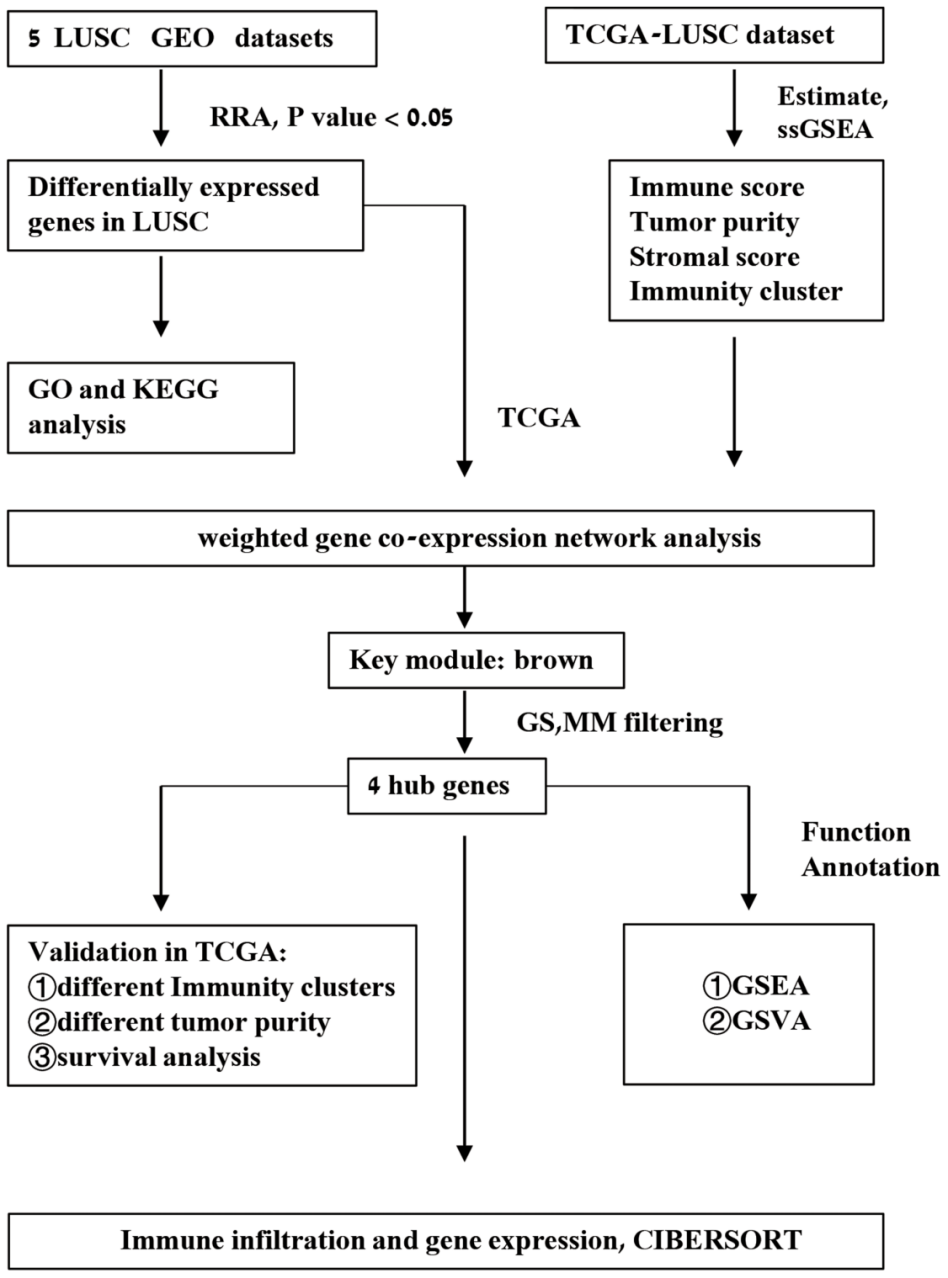
**Schematic representation of the study workflow.**

**Figure 2:**
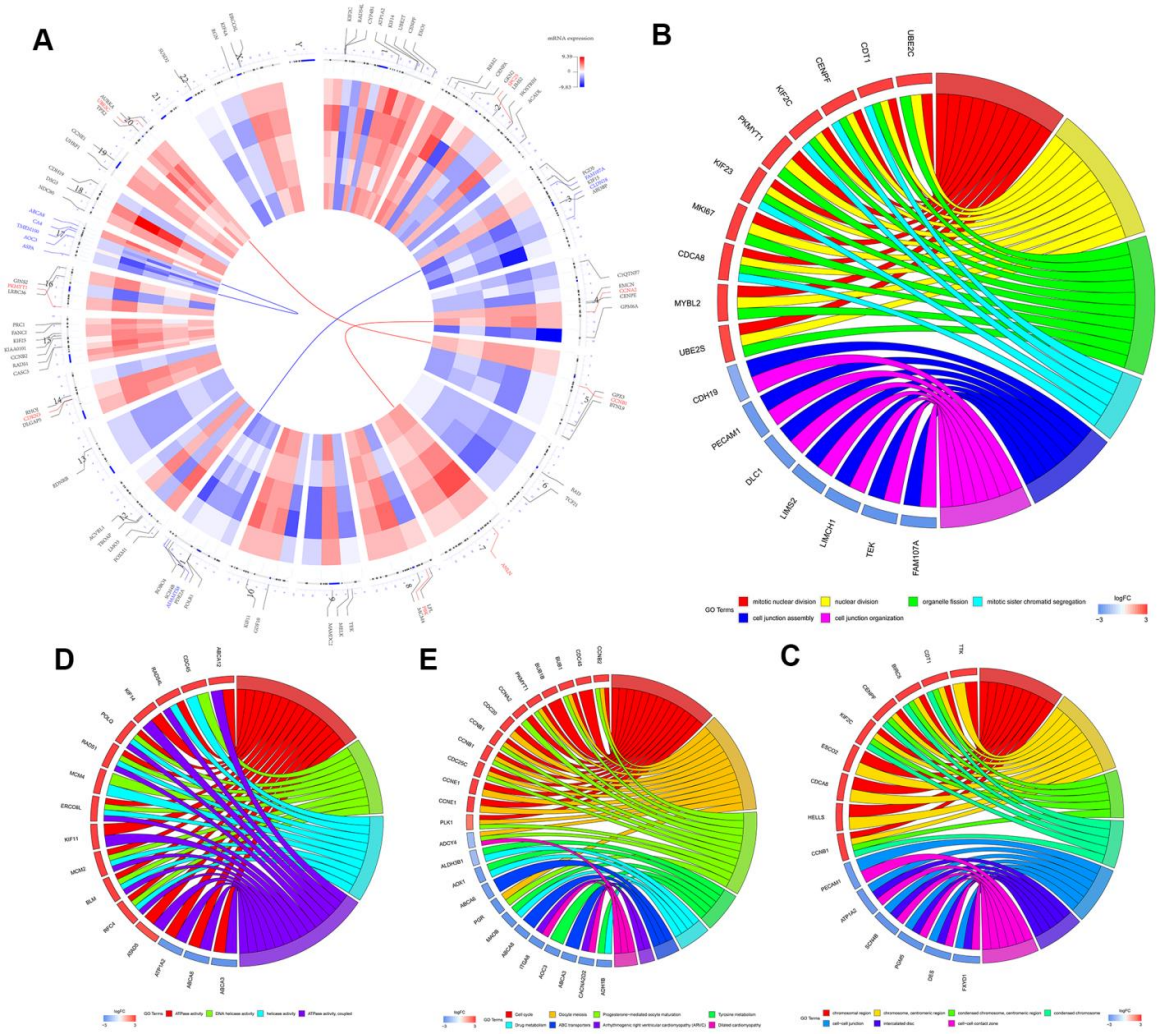
**Identification and functional enrichment analysis of robust DEGS in five GEO-LUSC datasets.** (**A**) The circular heatmaps show the differential expressed genes (DEGs) in the five GEO-LUSC datasets, which are shown in the inner circle. The upregulated genes are shown in red and the downregulated genes are represented in blue. Genes that are not present in a given dataset are shown in white. The outer circle represents the chromosomes. The lines indicate their specific chromosomal locations of each gene. The top 4 up-regulated and down-regulated genes according to the adjusted P values are shown in red and blue, respectively and are connected by the red and blue lines to the center of the circles. (**B**) The chord plot shows the relationship between the top 300 DEGs and the GO terms related to the biological processes (BP). (**C**) The chord plot depicts the relationship between the top 300 DEGs and the GO terms related to the cellular components (CC). (**D**) The chord plot depicts the relationship between the top 300 DEGs and the GO terms related to the molecular functions (MF). (**E**) The chord plot depicts the relationship between the top 300 DEGs and the KEGG pathways.

The study strategy for the identification, validation, and functional analysis of DEGs is shown in Figure 1. RRA analysis of the RNA-sequencing data from 5 eligible LUSC datasets (GEO database) identified 1738 DEGs (808 up-regulated and 930 down-regulated). The expression patterns and the chromosomal locations of the top 93 DEGs, visualized using the OmicCircos R package are shown in Figure 2A. The top 4 upregulated genes in the LUSC tissues were *ANLN, UBE2C, CCNA2,* and *CCNB1* and the top 4 downregulated genes in the LUSC tissues were *ASPA, FAM107A, ABCA8*, *and ADAMTS8*. The heatmap of the top 20 up-regulated and top 20 down-regulated DEGs are shown in Supplementary Figure 1.

We then performed GO and KEGG functional enrichment analyses of the top 300 DEGs. The top GO terms for the enriched biological processes (BP) were mitotic nuclear division, nuclear division, organelle fission, mitotic sister chromatid segregation, cell junction assembly, and cell junction organization (Figure 2B). The most significantly enriched GO term for cellular component (CC) was chromosomal region (Figure 2C). The top GO terms for the molecular functions (MF) were ATPase activity and DNA helicase activity (Figure 2D). The most enriched KEGG pathways were cell cycle, oocyte meiosis, and progesterone-mediated oocyte maturation (Figure 2E).

### Identification of immunity-related LUSC patient subtypes using ssGSEA and ESTIMATE

**Figure 3:**
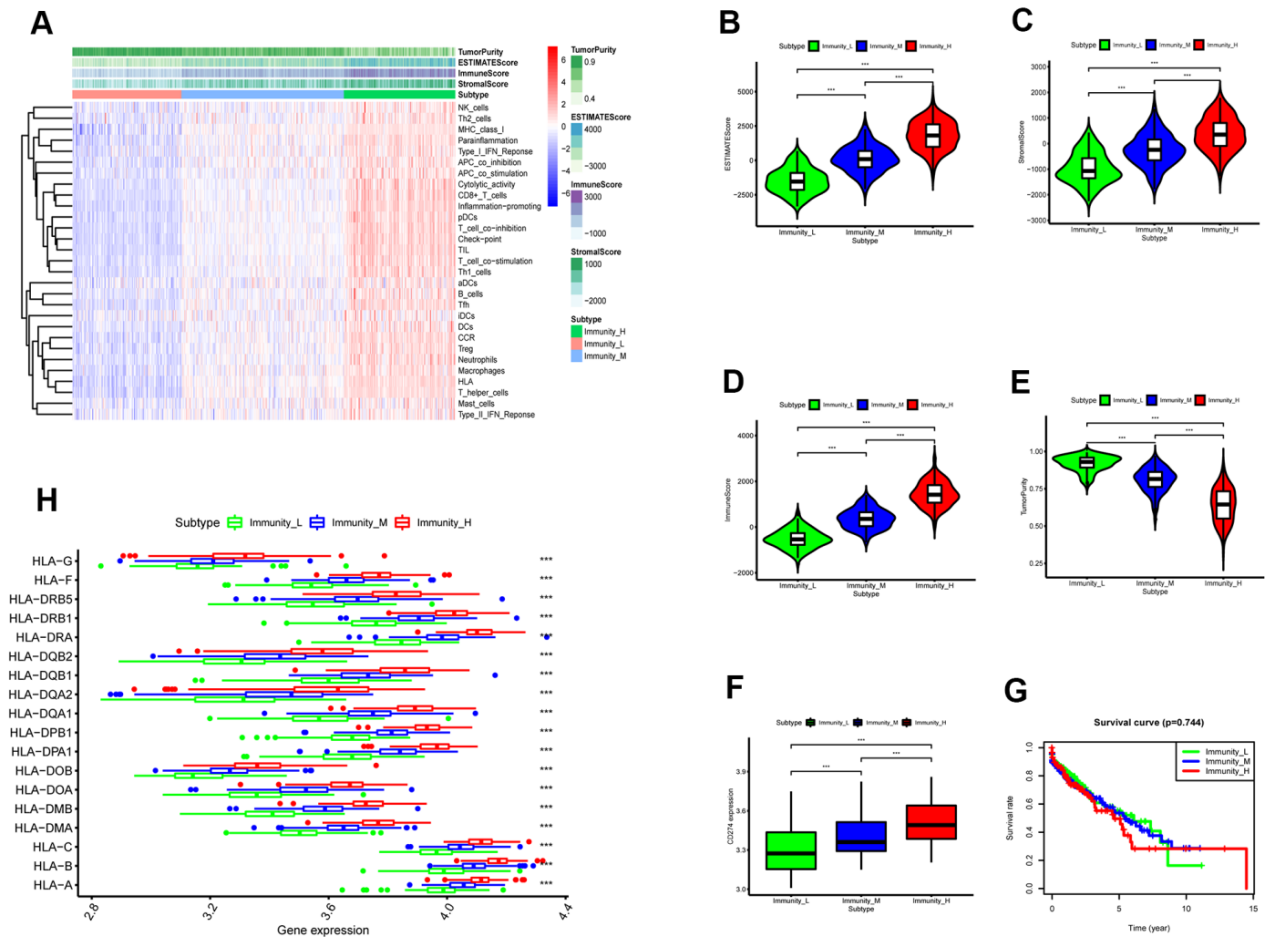
**ESTIMATE analysis of three immunity-related subtypes in the TCGA-LUSC samples based on ssGSEA scores.** (**A**) Hierarchical clustering of TGCA-LUSC samples based on the ssGSEA scores generated by analyzing the expression levels of the immunity-related gene sets. The data shows three distinct LUSC subgroups: high immunity, medium immunity, and low immunity. (**B**) ESTIMATE analyses of tumor purity, stromal scores, and immune scores of the high, medium, and low immunity groups of LUSC patient samples. Histogram plot shows the ESTIMATE scores of the three LUSC subgroups (Mann–Whitney U test, p<0.001). (**C**) Histogram plot shows the stromal scores of the three LUSC subgroups (Mann–Whitney U test, p<0.001). (**D**) Histogram plot shows the immune scores of the three LUSC subtypes (Mann–Whitney U test, p<0.001). (**E**) Histogram plot shows the tumor purity levels of the three LUSC subgroups (Mann–Whitney U test, p<0.001). (**F**) Histogram plot shows the PD-L1 expression levels of the three LUSC subgroups (ANOVA test, p<0.001).(**G**) Kaplan-Meier survival curve analysis shows the overall survival times of the LUSC patients belonging to the three LUSC subgroups (log-rank test: P>0.05). (**H**) Histogram plot shows the expression levels of HLA genes of the three LUSC patient subgroups (ANOVA, P<0.05). (**G**) Histogram plot shows the TOX expression levels of the three LUSC subgroups (ANOVA, P<0.05). Note: Immunity_H denotes high immunity group; Immunity_M denotes medium immunity group; Immunity_L denotes low immunity group.

We then analyzed the status of 29 immunity-related gene sets that represent diverse immune cell functions, and pathways using ssGSEA [13, 14, 17] in the TGCA-LUSC cancer samples (The numbers of these samples were in the Supplementary File 1) and ranked them according to ssGSEA scores. Hierarchically clustering of the TCGA-LUSC samples revealed high immunity, medium immunity, and low immunity subgroups (Figure 3A). The immune scores, stromal scores, and tumor purity of the three subtypes of LUSC samples were calculated using the ESTIMATE algorithm (Figure 3B). The high immunity group showed the highest stromal and immune scores and the lowest tumor purity whereas the low immunity group showed the lowest stromal and immune scores and the highest tumor purity (Kruskal–Wallis test: P< 0.001; Figure 3C–3E). The expression of *PD-L1* and *HLA* genes was highest in the high immunity group and lowest in the low immunity group (ANOVA test: P < 0.05; Figure 3F, 3H). This suggests that the high immunity group of LUSC patients might respond better to anti-*PD-L1* immunotherapy compared to the other two LUSC groups because *PD-L1* expression is positively associated with the anti-PDL1 immunotherapy response [18]. However, survival analyses showed no significant differences in overall survival times between the three LUSC patient groups (Figure 3G). This suggested that the LUSC cells escape immune surveillance despite the abundant presence of immune cells in the LUSC tissues.

**Figure 4:**
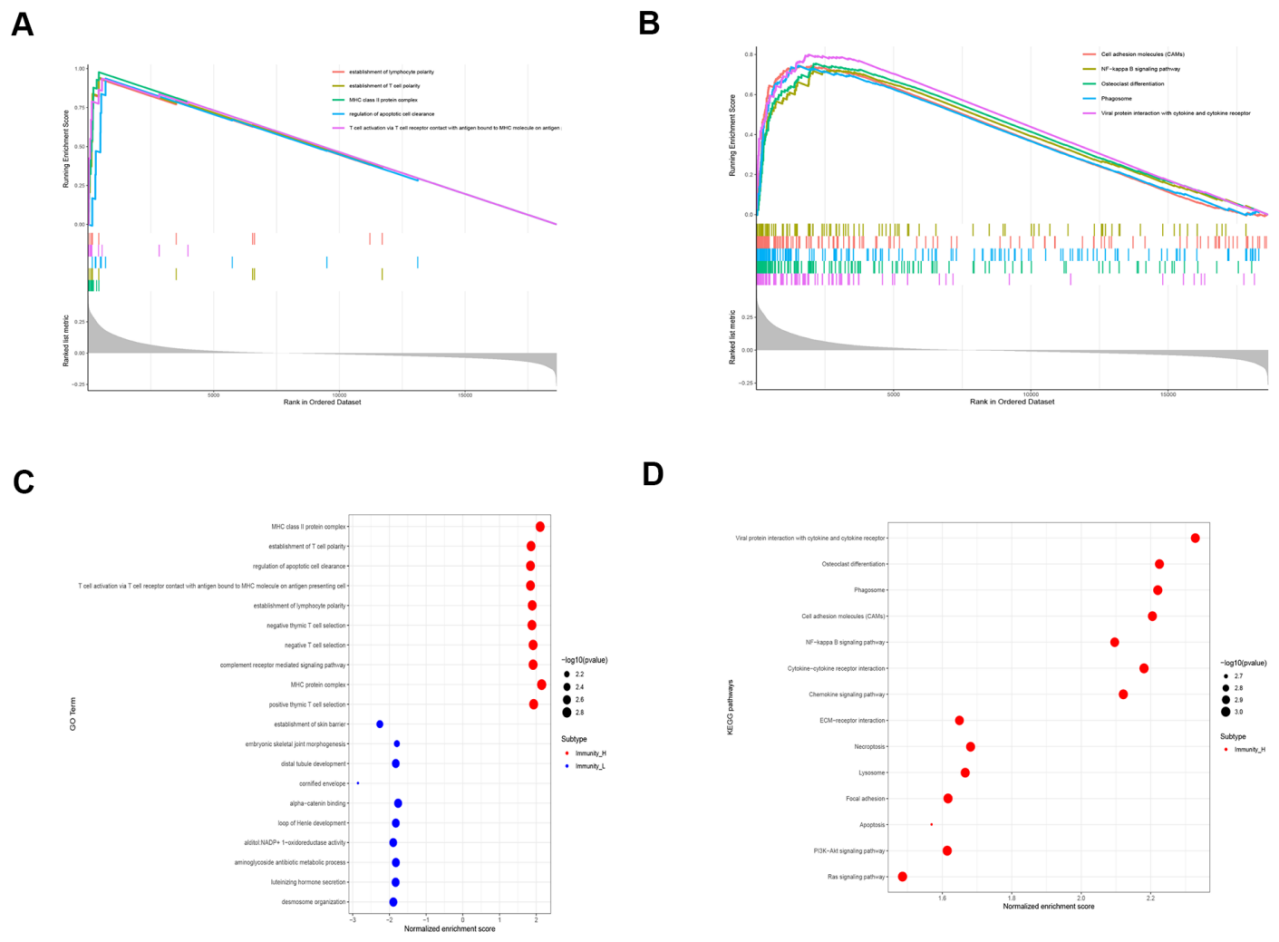
**Functional enrichment analyses of DEGS in the high immunity subgroup of LUSC patient samples.** (**A**) Gene set enrichment analysis (GSEA) results show the enriched GO terms and KEGG pathways in the high immunity subgroup of TGCA-LUSC samples. (**B**) The bubble plots show the enriched GO and KEGG pathways based on the analysis of upregulated genes in the high immunity subgroup of TGCA-LUSC samples.

We then performed GO and KEGG functional enrichment analyses of the DEGs between the high and low immunity groups of LUSC patient samples. The most significant GO terms and the KEGG pathways are shown in Figure 4A–4D. The high immunity LUSC group was enriched in several cancer-associated pathways such as NF-κB, PI3K–Akt, and RAS signaling pathways and immune signatures related to the establishment of lymphocyte and T-cell polarity.

### Identification of key modules associated with the immunity status of LUSC samples using WGCNA

**Figure 5:**
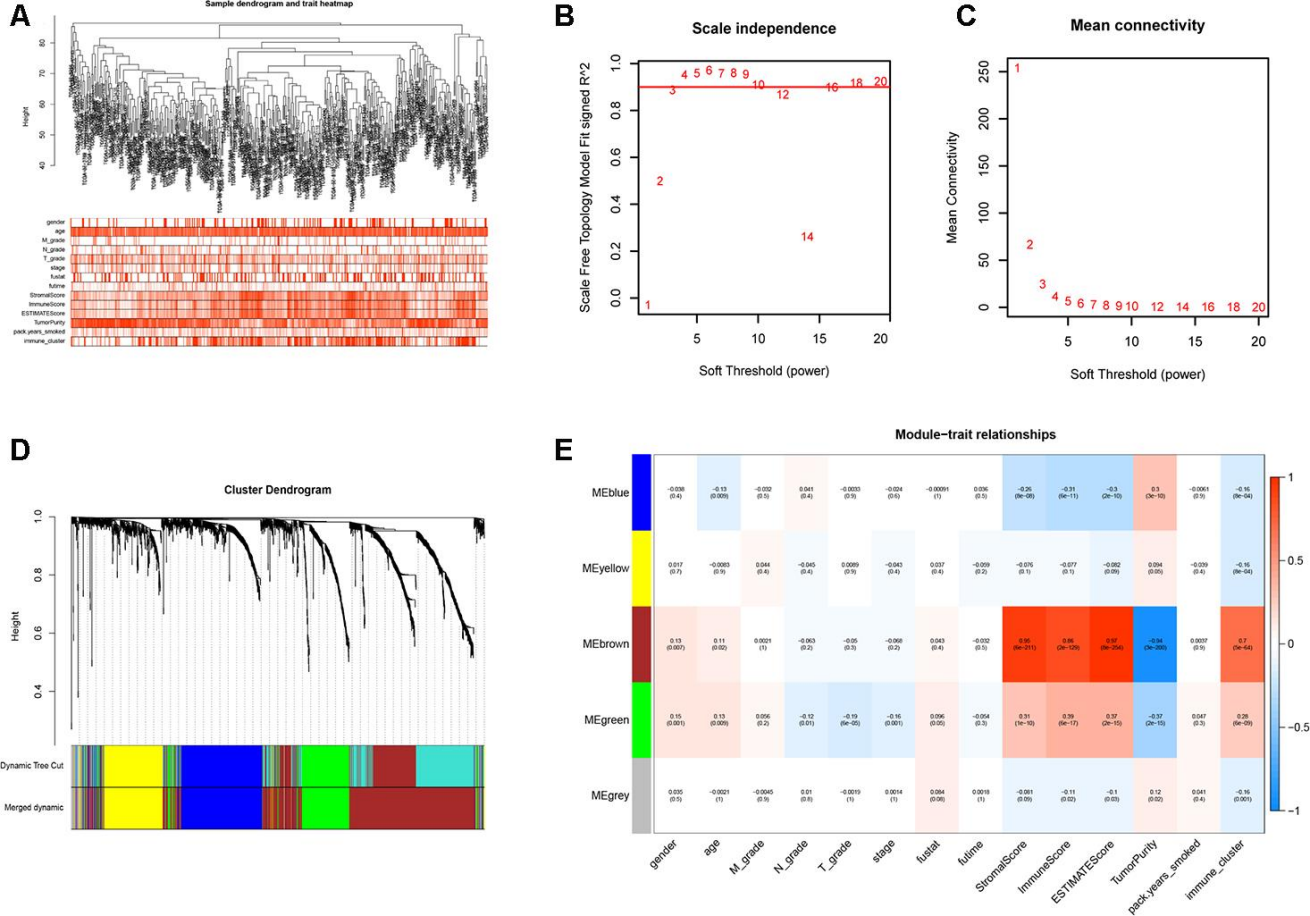
**Weighted gene correlation network analysis to identify key immunity-related gene modules in the TCGA-LUSC dataset and their correlation with the LUSC-related clinicopathological traits.** (**A**) The clustering dendrograms of robust DEGs identified by the RRA analysis in the TCGA-LUSC samples. The color intensity varies according to the clinicopathological characteristics such as age, TNM grades, stage and smoking history (smoking packs per year), immune scores, tumor purity, stromal scores and immunity subtypes (high, medium or low immunity subgroups). The red color indicates biochemical recurrence and white indicates absence of biochemical recurrence. For gender, red color denotes female and white color denotes male. (**B**, **C**) Network topology analyses for various soft-thresholding powers. The left panel shows the scale-free fit index (y-axis) as a function of soft-thresholding power (x-axis). The right panel shows the mean connectivity (degree, y-axis) as a function of soft-thresholding power. (**D**) The clustering dendrogram of all DEGs with dissimilarity measures based on topological overlap measure (TOM) together with assigned module colors. The non-clustering DEGs are shown in gray. (**E**) The heatmap shows the correlation between module eigengenes and the clinicopathological traits of LUSC. Each column contains the corresponding correlation coefficient and P value.

We performed WGCNA on the TCGA-LUSC dataset using the DEGs obtained from the RRA analysis to find key immunity-associated gene modules and evaluated their relationship with the clinicopathological characteristics of LUSC patients. The clinical information of LUSC patients including gender, age, TNM grades, stage, and smoking history was retrieved from the TCGA database. Figure 5A shows the clustering of the TCGA-LUSC samples according to their correlation with clinicopathological characteristics such as gender, age, TNM grades, stage, and smoking history, as well as the immune score, tumor purity, stromal score, and immunity-related LUSC patient groups (low-, medium- and high-immunity). We identified 5 immunity-related gene modules by setting the soft-threshold power as 4 (scale free R^2^ value = 0.96) and cut height value as 0.45 (Figure 5B–5D; non-clustering DEGs are shown in gray). The brown module showed the highest correlation with the immunity-related LUSC patient clusters (Pearson correlation coefficient = 0.7, P < 5E-64; Figure 5E).

**Figure 6:**
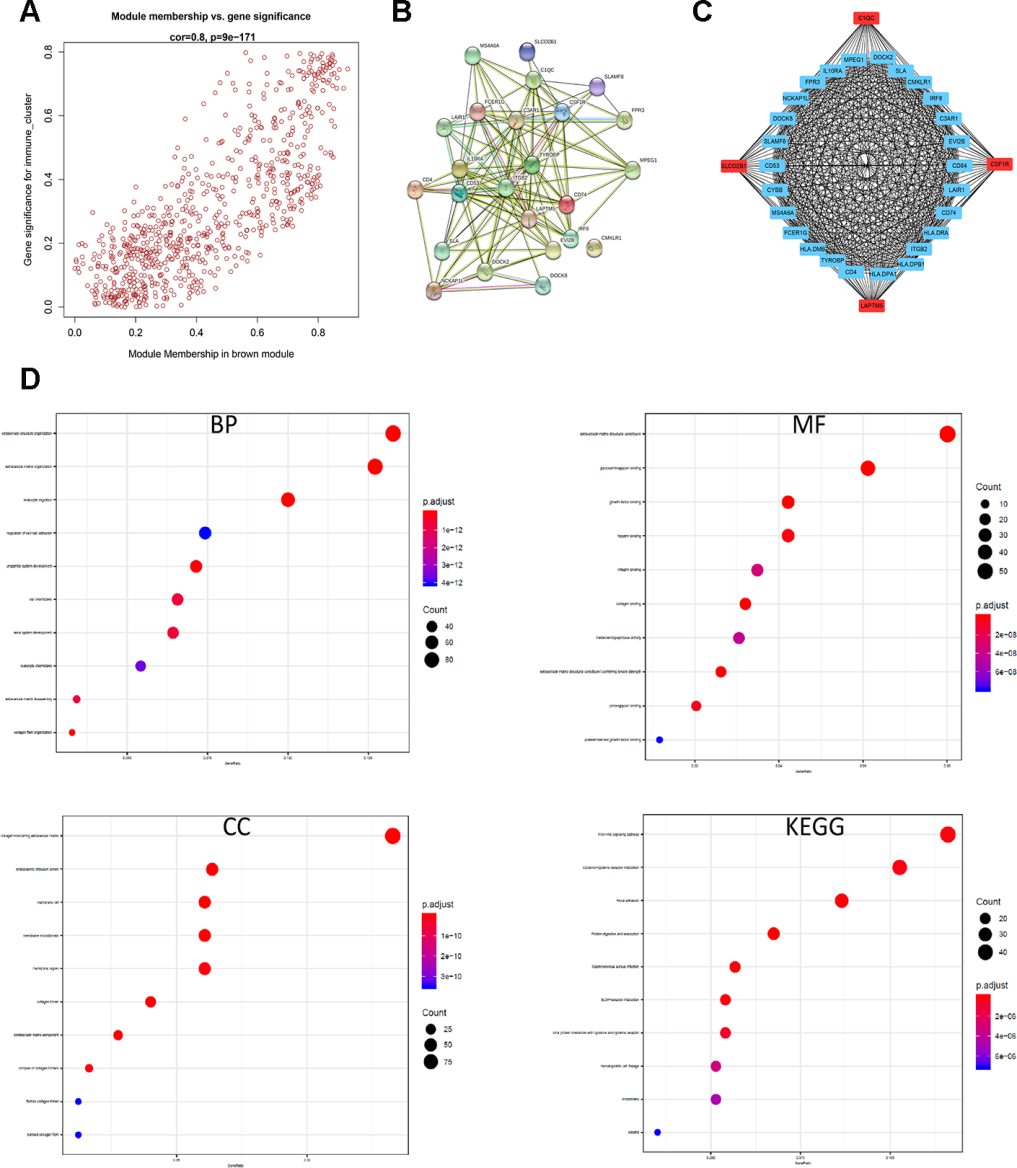
**Identification and functional annotation of the hub genes in the brown module.** (**A**) Scatter plot of the gene significance (GS) versus the module membership (MM) of the 30 hub genes in the brown module. (**B**) Protein-protein interaction (PPI) network of the 30 hub genes in the brown module. (**C**) Coexpression network analysis including visualization of the module membership (nodes) and the gene-gene connections (edges) of the top 30 hub genes in the brown module using the Cytoscape version 3.4.0 software (**D**) Functional enrichment analysis results show the enriched GO terms and KEGG pathways related to the DEGs in the brown module.

The brown module contains 762 genes (Figure 6A). Next, we calculated the connectivity within the brown module and selected the 30 most connected genes for further analysis, namely, *CD74, HLA.DRA, ITGB2, HLA.DPB1, LAPTM5, HLA.DPA1, CD4, C1QC, CSF1R, TYROBP, HLA.DMB, FCER1G, SLCO2B1, MS4A6A, CYBB, CD53, SLAMF8, DOCK8, NCKAP1L, FPR3, LAIR1, IL10RA, MPEG1, DOCK2, SLA, CMKLR1, IRF8, C3AR1, EVI2B and CD84*. Next, we generated the PPI interaction network and the coexpression network between the 30 genes (Figure 6B, 6C). GO and KEGG pathway analyses of the DEGs in the brown module show enrichment of gene sets related to extracellular structure organization and PI3K-Akt signaling pathway (Figure 6D).

### Validation of hub genes in the TCGA-LUSC dataset

**Figure 7:**
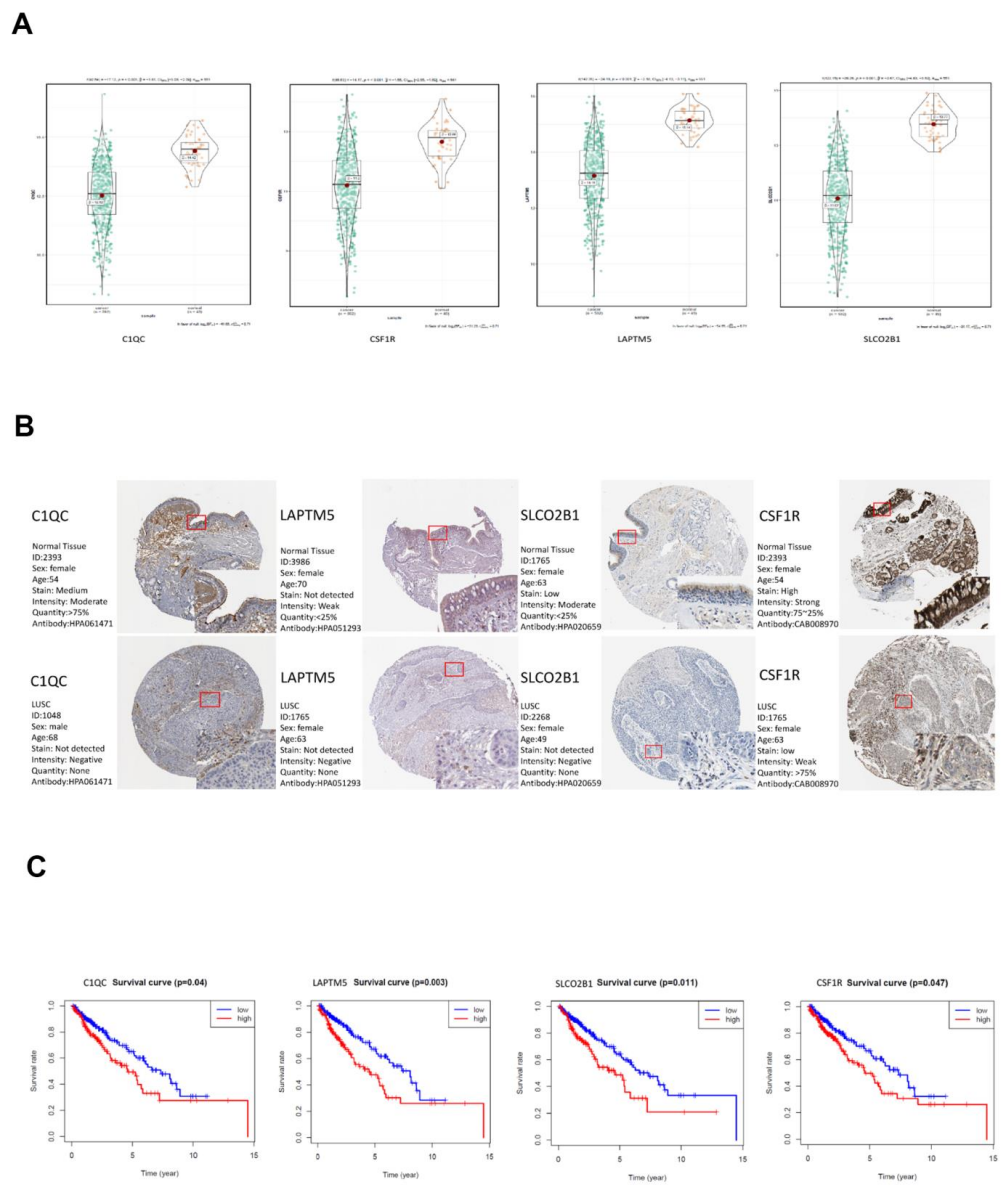
**Validation of the four hub genes in the TCGA-LUSC dataset.** (**A**) The expression levels of *LAPTM5, CSF1R, SLCO2B1* and *C1QC* mRNA in the TCGA-LUSC and adjacent normal lung tissue samples. (**B**) The expression levels of LAPTM5, CSF1R, SLCO2B1 and C1QC proteins in the LUSC and normal lung tissue samples based on the IHC data in The Human Protein Atlas database. (**C**) Correlation analysis of the mRNA expression levels of the 4 hub genes, *LAPTM5, CSF1R, SLCO2B1* and *C1QC* in the LUSC tissues and the overall survival time of the TCGA-LUSC patients. The red line indicates TCGA-LUSC samples with high expression of the 4 hub genes (above the best-separation value, n=157), and the blue line denotes the TCGA-LUSC samples with low expression of the 4 hub genes (below best-separation value, n=209).

Among the 30 hub genes in the brown module, we selected four genes, namely, *C1QC, CSF1R, LAPTM5* and *SLCO2B1* for validating the prognosis of LUSC patients and analyze their association with immune cell infiltration into LUSC tissues. The mRNA expression of all these 4 genes was significantly reduced in the LUSC samples compared to the adjacent normal lung tissue samples (P < 0.001; Figure 7A). The IHC data in the Human Protein Atlas database also showed that the expression of C1QC, CSF1R, LAPTM5 and SLCO2B1 proteins was significantly reduced in the LUSC tissues compared to the normal lung tissues (Figure 7B, Supplementary Table 1). Moreover, the mRNA expression of C1QC, CSF1R, LAPTM5 and SLCO2B1 was significantly higher in the high immunity group of LUSC patient samples compared to the low immunity group of LUSC patient samples (Supplementary Figure 2A). The mRNA expression of C1QC, CSF1R, LAPTM5 and SLCO2B1 was negatively associated with tumor purity (Supplementary Figure 2B). In addition, the mRNA data in the Cancer Cell Line Encyclopedia database showed that the expression of C1QC, CSF1R, LAPTM5 and SLCO2B1 mRNA was significantly lower in LUSC cell lines (n=23) compared to lymphoid cell lines (n=167) (Supplementary Figure 2C). Kaplan-Meier survival curve analysis showed that patients with higher expression of C1QC, CSF1R, LAPTM5 and SLCO2B1 were associated with worse overall survival times compared to those with lower expression of these four genes (Figure 7C).

**Table 1:** Characteristics of the included GEO-LUSC datasets.

**Data ID**	**Number of samples**		**GPLID**	**Number of rows per platform**
	**T**	**N**		
GSE21933	21	21	GPL6254	4815
GSE33479	14	27	GPL6480	19595
GSE33532	16	26	GPL570	14533
GSE62113	7	9	GPL14591	20818
GSE74706	8	18	GPL13497	21754

Note: GSE, Gene Expression Omnibus Series; GPL, Gene Expression Omnibus Platform; T, tumor samples; N, normal samples.

### Identification of LUSC patient clusters based on the expression of the Tox pathway genes

**Figure 8:**
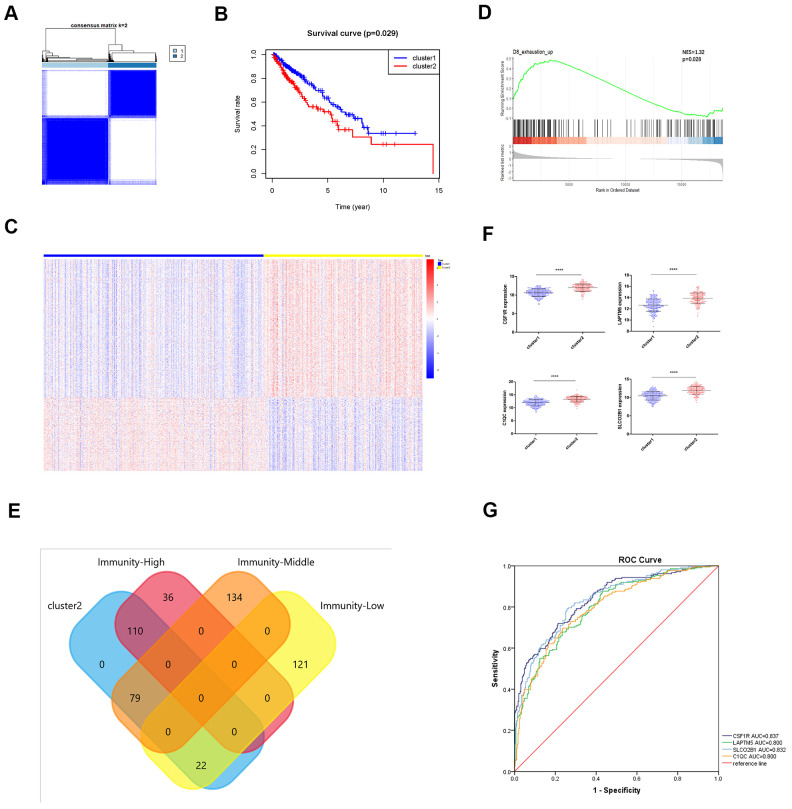
**Identification of immunity-based molecular subtypes of LUSC patient samples based on the expression of the TOX pathway gene signature.** (**A**) Clustering heat map shows the presence of two clusters among the TCGA-LUSC dataset based on the TOX pathway gene signature. (**B**) Kaplan–Meier survival curve analysis shows the differences in overall, survival times of cluster 1 and cluster 2 TCGA-LUSC patients. (**C**) The heatmap shows the expression of DEGs in the cluster 1 and cluster 2 TCGA-LUSC patients. (**D**) GSEA plot shows the upregulation of genes related to the exhausted CD8^+^ T cells in the cluster 2 TCGA-LUSC dataset compared to those in the cluster1 TCGA-LUSC dataset. The upregulated genes linked to the exhaustion of CD8^+^ T cells are shown on the left. Note: NES: normalized enrichment score. (**E**) Venn diagram shows the numbers of cluster 1 (n=36) and cluster 2 (n=110) molecular subtypes among the high immunity LUSC subgroup (n=146). (**F**) The histogram plots show the mRNA expression levels of *LAPTM5, CSF1R, SLCO2B1* and *C1QC* in the cluster 1 and cluster 2 LUSC samples. (**G**) ROC curve analysis shows the sensitivity and accuracy of the 4 hub genes, *LAPTM5, CSF1R, SLCO2B1* and *C1QC* to distinguish cluster 1 and cluster 2 samples based on their expression. The area under the ROC curve (AUC) values demonstrates that all 4 hub genes show high sensitivity and accuracy in distinguishing the LUSC patients belonging to the two clusters. Note: Immunity-High denotes high immunity group; Immunity-Middle denotes medium immunity group; Immunity-Low denotes low immunity group.

T cell exhaustion or hypo-responsiveness is observed during chronic infections and in solid tumors [19]. Recent studies demonstrate that the transcriptional regulator Tox plays an important role in T cell exhaustion [20–22]. The top 100 differentially upregulated genes in the *TOX*^WT^ CD8^+^ T cells compared to the *TOX*^∆^ CD8^+^ T cells in the GSE131643 dataset are defined as the *TOX* pathway gene signature [20] (These genes were shown in Supplementary File 2). We performed consensus clustering of the TCGA-LUSC samples based on the *TOX* pathway gene signature and identified two distinct clusters as shown in the clustering heatmap (Figure 8A). Kaplan-Meier survival analyses showed that the overall survival (OS) times were significantly lower in the cluster 2 LUSC patients compared to the cluster 1 LUSC patients (p=0.029; Figure 8B). The heatmap in Figure 8C shows the differentially expressed genes (DEGs) in cluster 2 compared to the cluster 1 LUSC samples. Furthermore, GSEA analysis using the exhausted versus effector CD8^+^ T cell data from the Molecular Signature Database for the GSE30962 dataset [23] showed that the density of exhausted CD8^+^ T cells was significantly higher in the cluster 2 LUSC samples compared to the cluster 1 LUSC samples (Figure 8D). Venn diagram shows that a higher proportion of high immunity group LUSC samples (110 of 146) belonged to the cluster 2 group (Figure 8E). Although survival analysis showed no significant differences in OS between the three LUSC subtypes (Figure 3G), *TOX* mRNA expression levels were significantly higher in the high immunity group of LUSC samples compared to the low immunity group (Supplementary Figure 3A). This suggests that TOX promotes CD8^+^ T cell exhaustion in the high immunity LUSC group. We also observed that *LAPTM5, C1QC, CSF1R, SLCO2B1* were highly expressed in the cluster 2 LUSC samples (Figure 8F). ROC curve analysis showed that all the four hub genes clearly distinguished the cluster 2 LUSC samples from the cluster 1 LUSC samples as shown by the AUC values (LAPTM5: AUC=0.800; C1QC: AUC=0.800; CSF1R: AUC=0.837; LCO2B1: AUC=0.832; Figure 8G).

### GSEA and GSVA reveal a close relationship between hub genes and immune dysfunction

**Figure 9:**
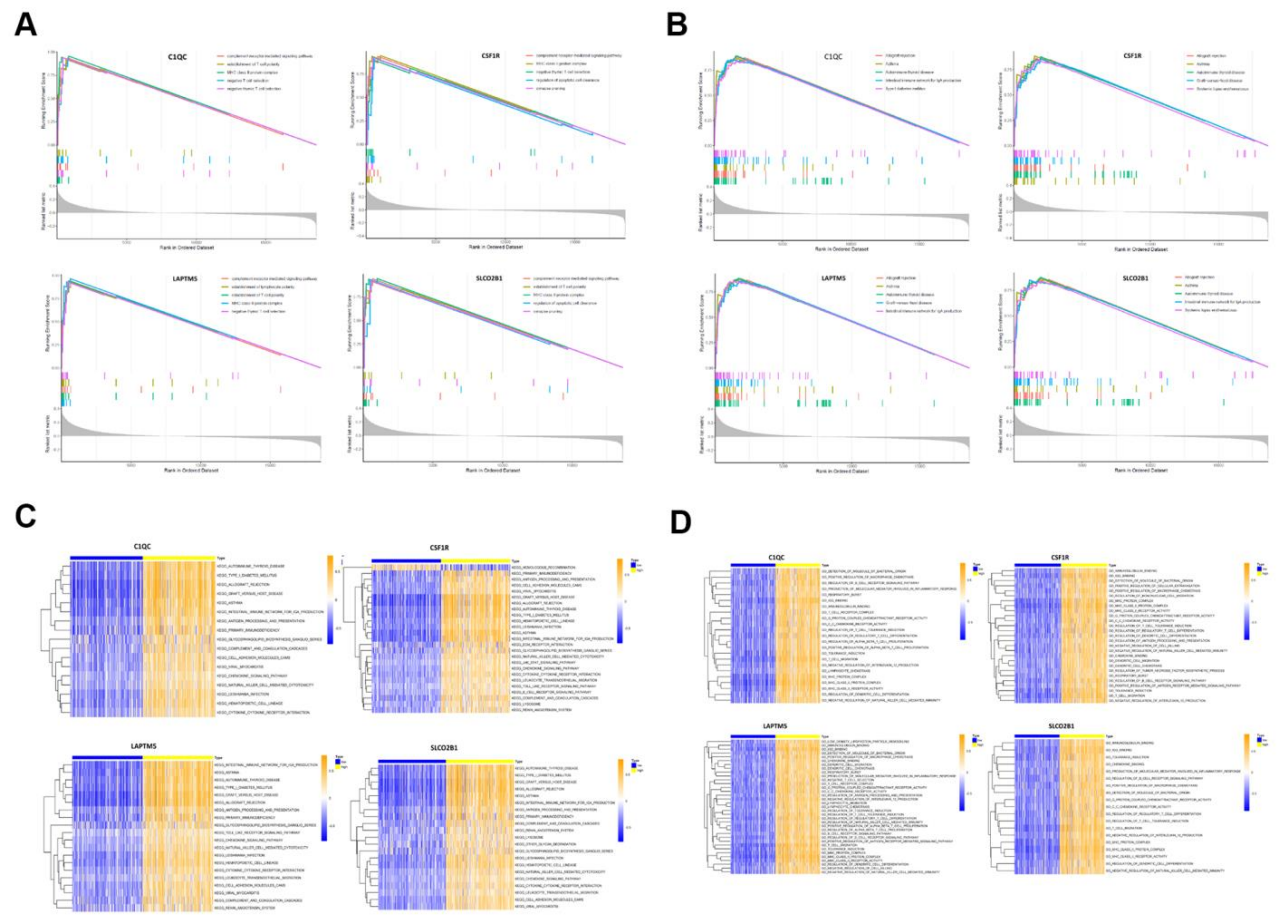
**Gene set enrichment analysis (GSEA) and gene set variation analysis (GSVA) of hub genes in the TCGA-LUSC dataset.** (**A**) The plot shows the enriched GO terms based on the GSEA enrichment score in the TCGA-LUSC patients with high expression of each of the four hub genes, *LAPTM5, CSF1R, SLCO2B1* and *C1QC*. (**B**) The plot shows the enriched KEGG pathways based on the GSEA enrichment score in the TCGA-LUSC patients with high expression of each of the four hub genes, *LAPTM5, CSF1R, SLCO2B1* and *C1QC*. (**C**) GSVA-derived clustering heatmaps show the enriched GO terms for the *LAPTM5, CSF1R, SLCO2B1* and *C1QC* in the TCGA-LUSC dataset. GO terms with log_2_ (foldchange) > 0.35 and adjusted P<0.05 are shown. (**D**) GSVA-derived clustering heatmaps show the enriched KEGG pathways for the *LAPTM5, CSF1R, SLCO2B1* and *C1QC* in the TCGA-LUSC dataset. KEGG signaling pathways with log_2_ (foldchange) > 0.2 and adjusted P<0.05 are shown.

We performed gene set enrichment analysis (GSEA) and gene set variation analysis (GSVA) of the TCGA-LUSC RNA-seq data to further investigate the potential functions of *LAPTM5, C1QC, SLCO2B1* and *CSF1R* in LUSC. The gene sets related to the complement receptor mediated signaling and MHC class II protein complex were significantly enriched in the LUSC patients with high *LAPTM5, C1QC, SLCO2B1* and *CSF1R* expression (Figure 9A). The gene set related to establishment of T cell polarity was enriched in the high-expression groups of *LAPTM5, C1QC* and *SLCO2B1*, whereas, the gene set related to negative thymic T cell selection was enriched in the *LAPTM5, C1QC* and *CSF1R* high-expression groups (Figure 9A). Gene sets related to the regulation of apoptotic cell clearance and synapse pruning were enriched in the *SLCO2B1* and *CSF1R* high-expression groups (Figure 9A).

GSVA results showed that gene sets related to negative T cell selection and establishment of lymphocyte polarity were enriched in the *C1QC* and *LAPTM5* high-expression groups (Figure 9C). GSVA confirmed GSEA results in the *LAPTM5, C1QC, SLCO2B1* and *CSF1R* high-expression groups (Figure 9C). GSVA results also showed that gene sets related to tolerance induction, negative regulation of natural killer cells mediated immunity, and negative regulation of *IL-12* production were enriched in the *LAPTM5, C1QC* and *CSF1R* high-expression groups (Figure 9C). The gene sets with the highest enrichment scores were all closely associated with the regulation of immunity in the tumor microenvironment. These findings suggest that the expression of immunosuppressive genes in the LUSC cells regulates the polarity of T cells and induces immune tolerance. KEGG pathway analysis results (Figure 9B) and GSVA results (Figure 9D) show that genes related to allograft rejection, asthma, and autoimmune thyroid disease pathways were enriched or upregulated in the *LAPTM5, C1QC, SLCO2B1* and *CSF1R* high expression groups (Figure 9B). GSVA results also showed that the gene set related to intestinal immune network for IgA production was enriched in the *LAPTM5, C1QC* and *SLCO2B1* high-expression groups (Figure 9D). Overall, the results showed that *LAPTM5, C1QC, SLCO2B1* and *CSF1R* high-expression groups were associated with immunosuppression in the LUSC tissues.

### Association of hub genes with immune infiltration

**Figure 10:**
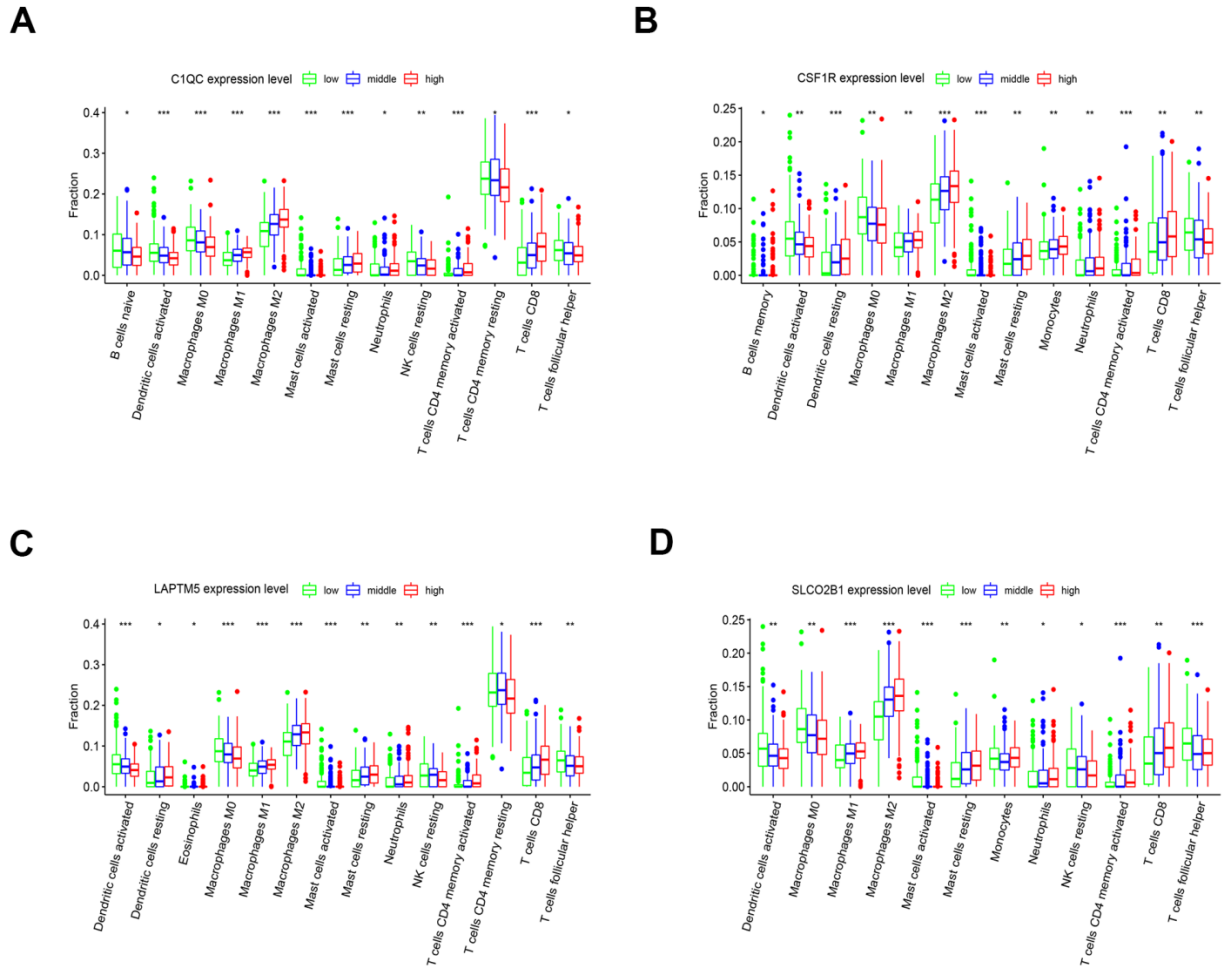
**The expression of the four hub genes is associated with differential infiltration of immune cells into the LUSC tissues.** (**A**–**D**) CIBERSORT analysis shows the association between infiltration of 22 immune cell types into the LUSC tissues and the expression levels of (**A**) *C1QC* (**B**) *CSF1R* (**C**) *LAPTM5* and (**D**) *SLCO2B1* genes. The LUSC patients were ranked into high, medium and low hub gene expression groups based on the levels of expression of each of the four hub genes, *C1QC, CSF1R, LAPTM5* and *SLCO2B1*. The red, blue, and green histograms indicate high, medium and low expression levels of the corresponding hub genes. The correlations between the groups were analyzed using Mann–Whitney U test.

The tumor microenvironment consists of the tumor cells, stromal cells, and the infiltrating immune cells. We utilized the CIBERSORT algorithm to investigate the association between the expression levels of hub genes and the infiltration of immune cells in the LUSC tissues. The expression levels of *LAPTM5, C1QC, SLCO2B1,* and *CSF1R* were all positively associated with the infiltration of M2 macrophages, M1 macrophages, resting mast cells and CD8^+^ T cells, and negatively associated with the infiltration of follicular helper T cells, M0 macrophages, and activated dendritic cells (Figure 10A–10D). Kaplan-Meier survival curves and log-rank tests showed that lower proportions of follicular helper T cells and M0 macrophages, and higher proportions of monocytes significantly correlated with poor overall survival LUSC patients (Supplementary Figure 4A). The correlation heatmap shows that the proportion of T follicular helper cells positively correlates with the proportions of CD8^+^ T cells, M1 macrophages and activated NK cells and negatively correlates with the proportions of the M2 macrophages (Supplementary Figure 4B).

## DISCUSSION

Immunotherapy has revolutionized the treatment of advanced lung squamous cell carcinoma (LUSC), but, the mechanisms that regulate immunity in the LUSC tissues are complex and not understood fully. For example, the overexpression of immune-effector cytokines such as IL-6 and PGE2 not only increase the tumor-infiltration of immune cells, but also induce stemness in the LUSC cells, thereby reducing their recognition by the immune surveillance machinery [8, 24]. In this study, we used bioinformatics analysis including RRA, WGCNA, ssGSEA, CIBERSORT and ESTIMATE to identify and characterize the hub genes associated with immune cell infiltration and the status of immunity in the LUSC tissues.

We first integrated and analyzed RNA-seq data from 5 eligible GEO-LUSC datasets using the RRA method and identified many robust DEGs such as *CCNA2* and *CCNB1,* which play a vital role in LUSC pathology [25]. Chromosome mapping showed that most of the top 93 DEGs were located on chromosome 1. Functional enrichment analyses of the DEGS showed up-regulation of genes related to nuclear division and cell cycle, which have been previously reported to play a role in LUSC development and progression [26]. Moreover, genes involved in cell junction assembly were significantly downregulated in the LUSC tissues, which influenced the proliferation and metastasis of breast cancer [27]. However, we did not observe significant enrichment of immune-related pathways in these DEGs using the GEO-LUSC data. Therefore, we analyzed the LUSC data using ESTIMATE algorithm and ssGSEA to determine differences in the immune cell infiltration in the TCGA-LUSC tumor samples. We identified high-, medium- and low-immunity subtypes among the TCGA-LUSC samples. The high immunity LUSC subtype was enriched in immunity-related gene signatures as well as cancer-associated *NF-κB*, *PI3K–Akt*, and *RAS* signaling pathways. However, survival analysis did not show any significant differences between these three subtypes, which suggested probable immunosuppressive mechanisms operating in the high immunity LUSC samples that suppress anti-tumor immunity despite having higher numbers of immune cells.

Previous studies have shown that prolonged exposure to tumor antigens induces exhaustion in CD8^+^ T cells, thereby attenuating their effector function and ability to identify and eliminate cancer cells; it also decreases their immunotherapeutic potential [23, 28]. T cell exhaustion is regulated by TOX and related proteins that are part of the TOX gene signature [19–21]. TOX mRNA levels were significantly elevated in the high immunity LUSC samples. Based on the expression of the *TOX* pathway genes, we identified two distinct clusters namely, cluster 1 and cluster 2. GSEA results showed that the proportions of exhausted CD8^+^ T cells were significantly higher in the cluster 2 LUSC samples compared to the cluster 1 LUSC samples. Kaplan Meier analysis also showed that the overall survival (OS) was significantly shorter for the cluster 2 LUSC patients compared to the cluster 1 LUSC patients. The Chi square test showed that 110 of the 146 high immunity group LUSC samples belonged to cluster 2. In fact, the proportion of cluster 2 samples was significantly higher in the high immunity LUSC samples compared to the medium immunity and low immunity LUSC samples (Supplementary Figure 3B). Overall, our results demonstrate that increased proportion of exhausted CD8^+^ T cells in the high immunity LUSC samples because of high TOX expression reduces overall survival to comparable levels in the medium and low immunity groups of LUSC patients.

We then used WGCNA to construct the co-expression network of the DEGs and identify hub genes that regulate immune function in the TGCA-LUSC patients. WGCNA results showed that the genes in the brown co-expression module correlate with the immune infiltration status of the TCGA-LUSC patient samples. GO and KEGG pathway analysis of genes in the brown module showed enrichment of genes related to extracellular structure organization and the PI3K-Akt signaling pathway. We further performed filtration of the genes in the brown module based on their GS and MM scores and identified 30 hub genes (*CD74, HLA.DRA, ITGB2, HLA.DPB1, LAPTM5, HLA.DPA1, CD4, C1QC, CSF1R, TYROBP, HLA.DMB, FCER1G, SLCO2B1, MS4A6A, CYBB, CD53, SLAMF8, DOCK8, NCKAP1L, FPR3, LAIR1, IL10RA, MPEG1, DOCK2, SLA, CMKLR1, IRF8, C3AR1, EVI2B, CD84*). We then investigated the prognostic value of four hub genes, namely, *LAPTM5, CSF1R, SLCO2B1* and *C1QC*, all of which have not been reported widely in literature.

Complement C1q C chain (*C1QC*) gene encodes the C-chain polypeptide of the complement C1q protein. C1QC protein is an integral part of the complement C1q protein as well as a key regulator of macrophage functions [29]. *LAPTM5* encodes the lysosomal-associated protein transmembrane 5 whose downregulation promotes the pathogenesis of LUSC [30]. Moreover, LAPTM5 activates NF-κB and MAPK signaling pathways in the macrophages, but negatively regulates T- and B- cell activation [31]. *CSF1R* gene encodes the colony stimulating factor 1 receptor protein. CSF1R inhibitor repolarizes M2 macrophages into the anti-tumorigenic M1 macrophages [32]. Not only that, a recent literature reported that treatment with anti-CSF1R preferentially depleted macrophages with an inflammatory signature but spared macrophage populations that in mouse and human expresses pro-angiogenic/tumorigenic genes [33]. *SLCO2B1* gene encodes a protein called the solute carrier organic anion transporter family member 2B1(OATP1). OATP2B1 mediates the uptake of indoxyl sulfate which amplifies macrophage activation via Dll4-Notch signaling [34]. Overall, the expression levels of *LAPTM5, CSF1R, SLCO2B1* and *C1QC* were all reduced in the LUSC tissue samples compared to the normal samples. Furthermore, expression levels of *LAPTM5, CSF1R, SLCO2B1* and *C1QC* were significantly higher in the high immunity group of LUSC patients compared to the low immunity group of LUSC patients. The expression of *LAPTM5, CSF1R, SLCO2B1* and *C1QC* was negatively associated with the tumor purity in the LUSC samples. In addition, the mRNA expression of C1QC, CSF1R, LAPTM5 and SLCO2B1 was significantly lower in LUSC cell lines compared to lymphoid cell lines. Based on these findings, we proposed that LAPTM5, CSF1R, SLCO2B1 and C1QC are mainly expressed in immune cells rather than LUSC cells in tumor samples. Moreover, LUSC patients with higher expression of *LAPTM5, CSF1R, SLCO2B1* and *C1QC* were associated with worse OS compared to those with lower expression of these genes. These findings suggest that *LAPTM5, CSF1R, SLCO2B1* and *C1QC* regulate the infiltration of immune cells into the tumor microenvironment in LUSC tissues. Besides, a previous study demonstrated that LAPTM5 downregulation is associated with LUSC progression [30]. *LAPTM5, C1QC, CSF1R,* and *SLCO2B1* were also highly expressed in the cluster 2 LUSC samples. The ROC curve analysis showed that all the four genes accurately distinguished cluster 1 and cluster 2 LUSC samples. The AUC values for LAPTM5, C1QC, CSF1R, and SLCO2B1 were 0.800, 0.800, 0.837, and 0.832, respectively. This suggests that these four hub genes were strongly associated with CD8^+^ T cell exhaustion. We then performed GSEA and GSVA to determine the immunity-related functions of the 4 hub genes. The GO and KEGG pathway analysis of high-hub gene expressing LUSC tissues showed enrichment of gene sets related to autoimmune diseases such as asthma and autoimmune thyroid disease, establishment of T cell polarity, negative thymic T cell selection, negative thymic T cell selection, and the MHC class II protein complex. This suggests that the four hub genes alter the functions of the monocytes and macrophages and stimulate the differentiation of T cells in the tumor immune microenvironment.

We used the CIBERSORT algorithm to determine the proportions of 22 different types of immune cells in the LUSC microenvironment [16]. The results showed that the expression levels of *LAPTM5, CSF1R, SLCO2B1* and *C1QC* were positively associated with the proportions of CD8^+^ T cells, M1 macrophages, and M2 macrophages, and negatively associated with the proportions of activated dendritic cells, M0 macrophages, and follicular helper T cells. This suggests that the high expression of the 4 hub genes promotes differentiation of M0 macrophages into M1 and M2 macrophages. Kaplan-Meier survival curves and log-rank tests showed that lower proportions of follicular helper T cells correlates with short overall survival of LUSC patients, which is similar to previously reported findings [35]. Furthermore, the proportion of follicular helper T cells positively correlates with the proportion of CD8^+^ T cells, M1 macrophages, and activated natural killer (NK) cells and negatively correlates with the proportions of M2 macrophage cells. These results further confirm that the follicular helper T cells mediate the anti-tumor effects in the tumor microenvironment. The GSVA and GSEA results demonstrate that the high expression of these 4 hub genes contributes to differential infiltration of different types of immune cells and also promotes an immunosuppressive microenvironment in the LUSC tumors.

Our study has some limitations. Firstly, we did not perform *in vivo* and *in vitro* experiments to validate the findings regarding the 4 hub genes in LUSC immunity. Secondly, we did not explore several other hub genes that are related to immunity but did not show aberrant expression in the LUSC tissues. Therefore, future in-depth investigations are necessary to confirm our findings.

In conclusion, we combined RRA, WGCNA, CIBERSORT, and other bioinformatics tools to identify four hub genes, *LAPTM5, CSF1R, SLCO2B1* and *C1QC* that are strongly associated with the infiltration of different types of immune cells into the LUSC tissues and influence patient survival outcomes. Functional enrichment analyses with GSEA and GSVA demonstrates that high expression of these 4 hub genes is associated with immunosuppression in the LUSC tissue microenvironment and CD8^+^ T cell exhaustion. Further investigations are necessary to validate the potential of these 4 hub genes as immunotherapy targets for LUSC patients.

## MATERIALS AND METHODS

### Selection of LUSC gene expression datasets

We queried the Gene Expression Omnibus (GEO) database to identify LUSC patient tissue datasets with normal lung tissue samples as controls with adequate information regarding technologies and platforms used for high throughput gene expression analysis. Based on these criteria, we downloaded five LUSC datasets from the repository (The names of the five datasets were shown in Table 1). The Human Protein Atlas (http://www.proteinatlas.org) database (HPAD) was used to validate the expression of LAPTM5, CSF1R, C1QC, and SLCO2B1 proteins in LUSC tissues based on immunohistochemistry. The links for HPAD are shown in Supplementary Table 1.

The Cancer Cell Line Encyclopedia database (https://portals.broadinstitute.org/ccle/about) was used to validate the expression of LAPTM5, CSF1R, C1QC, and SLCO2B1 mRNA in LUSC cell lines and lymphocytic cell lines.

### Identification of robust DEGs using RRA

We downloaded the transcriptome files of the 5 GEO datasets, normalized the data, and identified the DEGs between LUSC and normal lung tissue samples using the limma R package [36]. We then identified the most significant DEGs by integrating the 5 datasets using RRA [12]. The genes were then ranked according to their P values and those with adjusted P < 0.05 were considered as robust DEGs in the RRA analysis. We then visualized the expression patterns and the chromosomal locations of the top 47 up-regulated and top 46 down-regulated genes (n=93) using the OmicCircos R package.

### Hierarchical clustering of TGCA-LUSC samples based on ssGSEA scores of 29 immunity-related gene set signatures

We first determined the single-sample gene-set enrichment analysis (ssGSEA) score [13, 14] based on the expression levels of 29 immunity-related gene set signatures for each smaple in the TCGA-LUSC dataset (Supplementary File 1). Then, we performed hierarchical clustering of the LUSC samples according to ssGSEA scores and classified the LUSC samples into high, medium, and low immunity subgroups.

### ESTIMATE and CIBERSORT analysis of the LUSC samples

We determined the immune score (immune cell infiltration levels), tumor purity, and stromal score (stromal content) for each of the LUSC samples using ESTIMATE [15]. We used CIBERSORT [16] to calculate the proportions of 22 human immune cell subsets in each of the TCGA-LUSC tumor samples using 1000 permutations and P< 0.05 as the criteria. We then ranked the TCGA-LUSC tumor samples according to the expression levels of each of the 4 hub genes. We classified the LUSC samples into three groups, namely, low-, medium- and high-expression groups based on the expression of each of the hub genes and compared the proportions of the 22 different immune cell subsets between the high- and low-expression groups using the Mann–Whitney U test.

### Function enrichment analyses

The Gene Ontology (GO) and Kyoto Encyclopedia of Genes and Genomes (KEGG) pathway enrichment analyses of the DEGs were performed using the clusterprofiler R package [37]. The GO terms or KEGG pathways with adjusted P <0.05 were considered statistically significant and visualized using the GO plot R package [38].

### WGCNA

We extracted the top 6748 up-regulated DEGs in the LUSC samples from the RRA analysis and the gene expression data for the TCGA-LUSC dataset. We then analyzed the association between the clinicopathological traits and the 4 hub genes using the WGCNA R package [39]. The adjacency matrix was transformed into a topological overlap matrix (TOM) and the DEGs were sorted into different gene modules according to the TOM-based dissimilarity measure. The key gene modules were identified by setting the soft-thresholding power as 4 (scale free R^2^ = 0.96), cut height as 0.45, and minimal module size as 30. The module that showed the highest correlation with the clinical traits was subjected to functional enrichment analysis (GO and KEGG analyses) to determine the biological functions of the genes and also used to screen the hub genes. The hub genes were defined as those with gene significance (GS) > 0.3 and a module membership (MM) > 0.8. The module membership (nodes) and the gene-gene connections (edges) of the top 30 hub genes were extracted from the topology overlay matrix and used to construct coexpression networks with the Cytoscape software version 3.4.0 [40]. The PPI network of DEGs was analyzed by the STRING database (http://string-db.org).

### Validation and survival analysis of hub genes

We used the ggstatsplot R package (https://cran.rproject.org/web/packages/ggstatsplot/) to validate the expression levels of the hub genes in the LUSC and adjacent normal lung tissue samples. We also evaluated their correlation with the clinicopathological features in the TCGA-LUSC dataset. We analyzed the data using the t-test or one-way analysis of variance (ANOVA). We also performed survival analysis for the hub genes in the TCGA-LUSC dataset using the survminer R (https://CRAN.R-project.org/package=survminer) and survival R (https://CRAN.R-project.org/package=survival) packages. The tumor samples in the TCGA-LUSC dataset were divided into two groups based on the median expression cut-off value for each hub gene and then Kaplan-Meier (K-M) survival curves were constructed.

### Gene set enrichment analysis (GSEA) and gene set variation analysis (GSVA)

We performed GSEA analysis of the hub genes using the TCGA-LUSC dataset with the clusterprofiler R package [37]. The GSVA R package was used to determine the pathways associated with the hub genes [14]. The LUSC patients were ranked according to the expression level of each hub gene into low-, medium-, and high-expression groups. P < 0.05 was considered as statistically significant. We downloaded the gene set “c2.cp.kegg.v6.2.symbols.gmt”and“c5.all.v6.2.symbols.gmt” from the Molecular Signature Database (http://software.broadinstitute.org/gsea/msigdb/index.jsp) as the reference gene set.

### Identification of immunity-related molecular subtypes of LUSC patients

The expression profiles of the immunity-related genes in the TCGA-LUSC cohort were used to identify the LUSC subtypes. We downloaded the GSE131643 dataset [22] from the Gene Expression Omnibus database (http://www.ncbi.nlm.nih.gov/geo/) and identified the human Entrez IDs using the clusterProfiler R package version 3.8.1 [37] for the highly expressed genes with a FDR P value < 0.05 and a log_2_FC > 0.15 when compared with the TOX pathway gene signature. The TOX gene signature includes 100 TOX pathway-related genes (Supplemental File 2) obtained previously by comparing TOX^wt^ CD8^+^ T cells vs. TOX^Δ^ CD8^+^ T cells. Therefore, we evaluated the molecular subtypes of the LUSC patients in the TCGA cohort using the ConsensusClusterPlus R package [41] using default parameters for the classification and the total numbers of cluster numbers as 2. The analysis of differently expressed genes (DEGs) was performed with the limma R package [36]. An empirical Bayesian method was applied to estimate the fold change between clusters 1 and 2 using moderated t-tests. The genes with adjusted P-value < 0.05 and absolute log_2_FC > 1.5 were denoted as DEGs. We used the clusterprofiler R package to perform the GSEA analysis [37]. The “ACUTE_VS_CHRONIC_LCMV_PRIMARY_IARY_INF_CD8_TCELL_UP” gene set was downloaded from the Molecular Signature Database (http://software.broadinstitute.org/gsea/msigdb/index.jsp) and selected as the reference gene set. This gene set is from the GSE30962 dataset that has been previously used to assess the exhaustion of CD8^+^T cells [20, 21, 28]. The survival curve analysis was performed using the R survival R package. The receiver operating characteristic (ROC) curves were used to determine the diagnostic value of the hub genes and the area under the ROC curve (AUC) values were calculated using the IBM SPSS Statistics 21.0 software. The Venn diagram was plotted using the FunRich software version 3.1.3.

## Supplementary Material

Supplementary Figures

Supplementary Table 1

Supplementary File 1

Supplementary File 2
