# Supplementary material for: Four hub genes regulate tumor infiltration by immune cells, antitumor immunity in the tumor microenvironment, and survival outcomes in lung squamous cell carcinoma patients
Source: Aging (Albany NY). 2021 Jan 10;13(3):3819–42. doi: 10.18632/aging.202351 (PMC7906216; doi:10.18632/aging.202351)
Supplement: Supplementary Figures [file aging-13-202351-s001.pdf]

SUPPLEMENTARY FIGURES

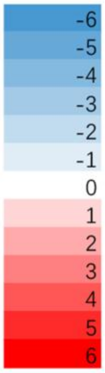

	GSE33479	GSE74706	GSE33532	GSE62113	GSE21933
ANLN	1.981559	5.68401	4.120976	1.737412	3.387262
UBE2C	2.415897	5.445347	3.872097	1.973665	1.124623
CCNA2	2.411964	3.932987	2.914136	1.591579	3.005617
CCNB1	1.148046	3.414343	3.487916	1.583203	2.21739
ASPM	3.328311	4.461253	2.726073	1.770178	3.706386
SPC25	2.318447	4.60277	3.038911	1.725514	2.520763
PKMYT1	2.139416	4.081931	1.394654	2.325167	1.975907
CDKN3	2.24673	3.84079	3.338855	2.209311	3.095467
PBK	1.946117	4.657478	4.493581	2.027653	3.201097
CEP55	1.965645	4.710963	3.941404	2.281531	4.199844
FANCI	1.863464	3.273734	2.280176	1.532925	2.010792
CCNE1	2.175195	3.207143	1.909297	1.614696	1.788673
ZWINT	1.859488	3.79175	2.988989	2.225372	2.726301
UBE2T	2.004286	4.179788	3.724696	-0.33685	3.334989
TROAP	2.384981	4.309073	2.024781	2.855772	2.86355
TPX2	2.661773	4.781612	3.989509	1.228921	3.438778
CENPF	4.152262	4.717754	2.853536	3.128233	2.563727
RAD51	3.204365	3.906229	2.448679	1.326683	2.601716
KIF11	1.867276	3.475967	3.085652	2.277222	2.777182
NUF2	3.464915	3.730721	3.546208	1.69601	3.226264
ASPA	-1.32803	-4.77463	-2.83283	-2.73983	-2.94239
FAM107A	-1.23312	-5.68669	-3.88925	-2.96917	-5.63438
ABCA8	-1.35263	-4.03614	-5.23121	-2.43525	-2.88501
CCDC48	-1.07229	-2.46173	NA	-3.39265	-3.5829
ADAMTS8	-1.33302	-6.31223	-3.86146	-1.55761	-4.53288
CA4	0.094877	-6.10659	-4.15477	-5.02182	-5.51937
AOC3	-1.39418	-4.27612	-2.9717	-1.29505	-2.58277
CLDN18	-0.59136	-8.38639	-4.9066	-2.65184	-5.30737
TMEM100	-1.55615	-6.71354	-5.51338	-2.69398	-4.75006
SOD3	-1.68326	-2.58358	-1.05512	-2.89697	-2.54146
FMO2	-1.60809	-5.03954	-3.20408	-3.58352	-2.73419
EMCN	-1.16569	-4.19885	-4.07978	-2.52077	-2.96645
ADH1B	-1.39313	-6.43066	-6.03131	-4.03508	NA
LIMS2	-1.23341	-3.58125	-1.94004	-2.2148	-2.86662
NOSTRIN	-1.51198	-3.92272	-3.66004	-2.27118	-3.25624
ABCA6	-1.57883	-2.77361	-2.22674	-2.35319	-2.01423
TCF21	-1.40514	-5.28304	-3.93268	-2.61819	-0.56001
ACADL	-0.89485	-4.23782	-3.60873	-3.48619	-3.37118
LRRC36	-1.34381	-5.45997	-3.59754	-1.93117	-4.66586
LPL	1.485231	-4.30958	-3.86026	-2.11104	-3.75505

Supplementary Figure 1. The circular visualization of the expression patterns of the top 93 DEGs and their chromosomal positions. The heatmap shows the top 20 up-regulated genes and top 20 down-regulated genes according to their P value. The columns represent each of the five GEO datasets. Each row represents a single gene. Red and blue indicate up-regulation and down-regulation, respectively. The numbers in the heatmap indicate log (fold change) values in each dataset as calculated by the limma R package.

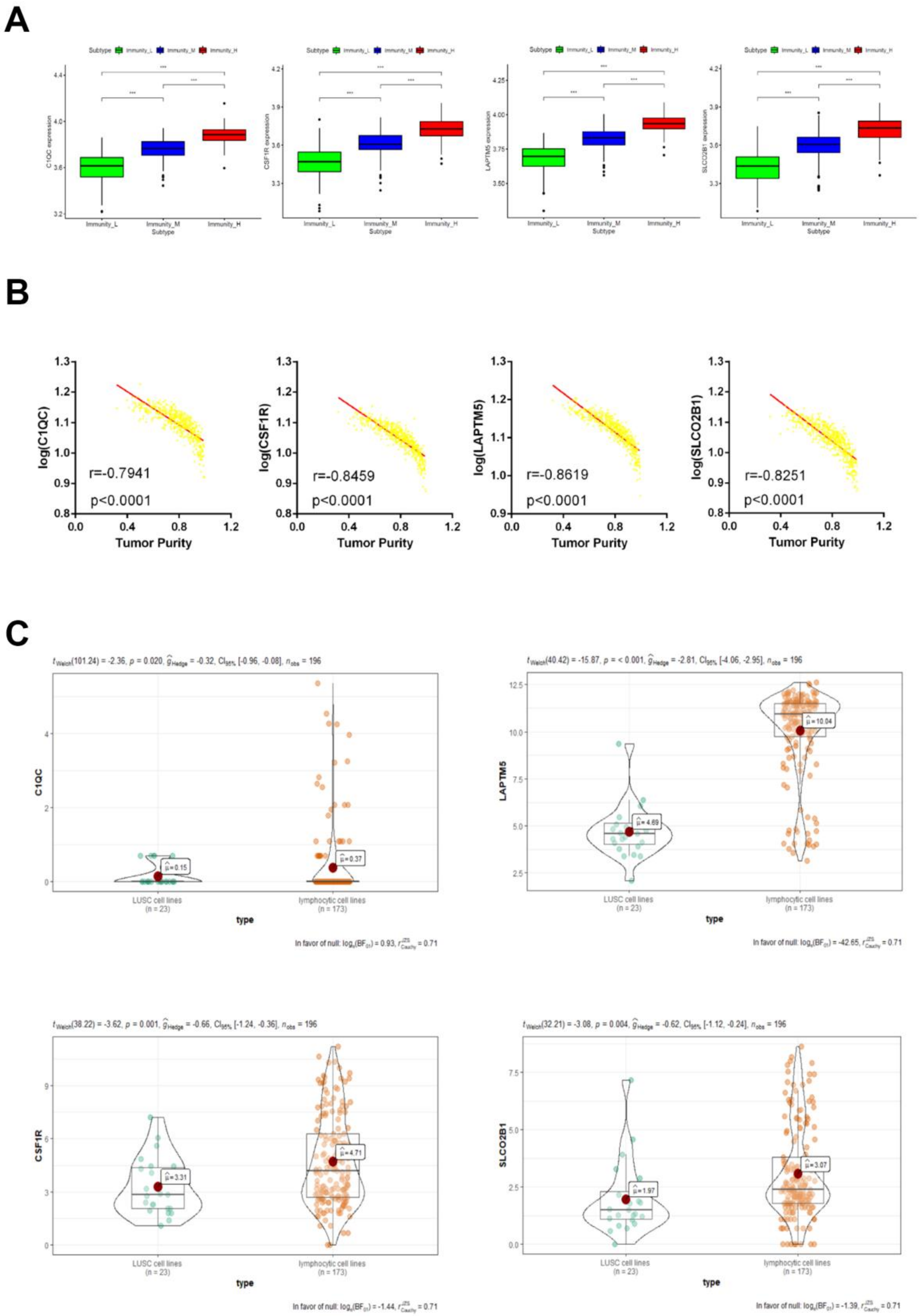

Supplementary Figure 2. Validation of the hub genes in the TCGA-LUSC dataset. (**A**) The histogram plots show the mRNA expression of *LAPTM5, CSF1R, SLCO2B1* and *C1QC* in the high, medium and low immunity LUSC subgroups. (**B**) The histogram plot shows the mRNA expression levels of *LAPTM5, CSF1R, SLCO2B1* and *C1QC* in the LUSC samples with different tumor purity levels. (**C**) The expression levels of *LAPTM5, CSF1R, SLCO2B1* and *C1QC* mRNA in the LUSC cell lines(n=23) and lymphocytic cel lines(n=167).

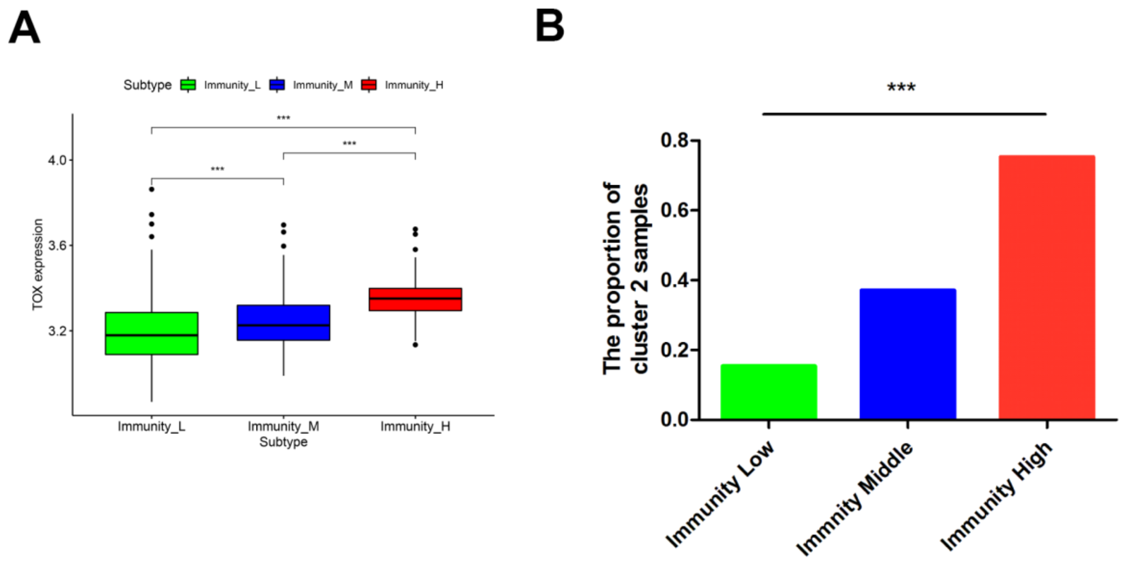

Supplementary Figure 3. (**A**) The histogram plots show the expression levels of *TOX* in the high, medium and low immunity LUSC subgroups (ANOVA test, $p<0.001$). (**B**) Comparison of the proportions of cluster 2 samples in the high, medium and low immunity LUSC subgroups (Chi-square test, $p<0.001$). Note: Immunity-High denotes high immunity group; Immunity-Middle denotes medium immunity group; Immunity-Low denotes low immunity group.

A

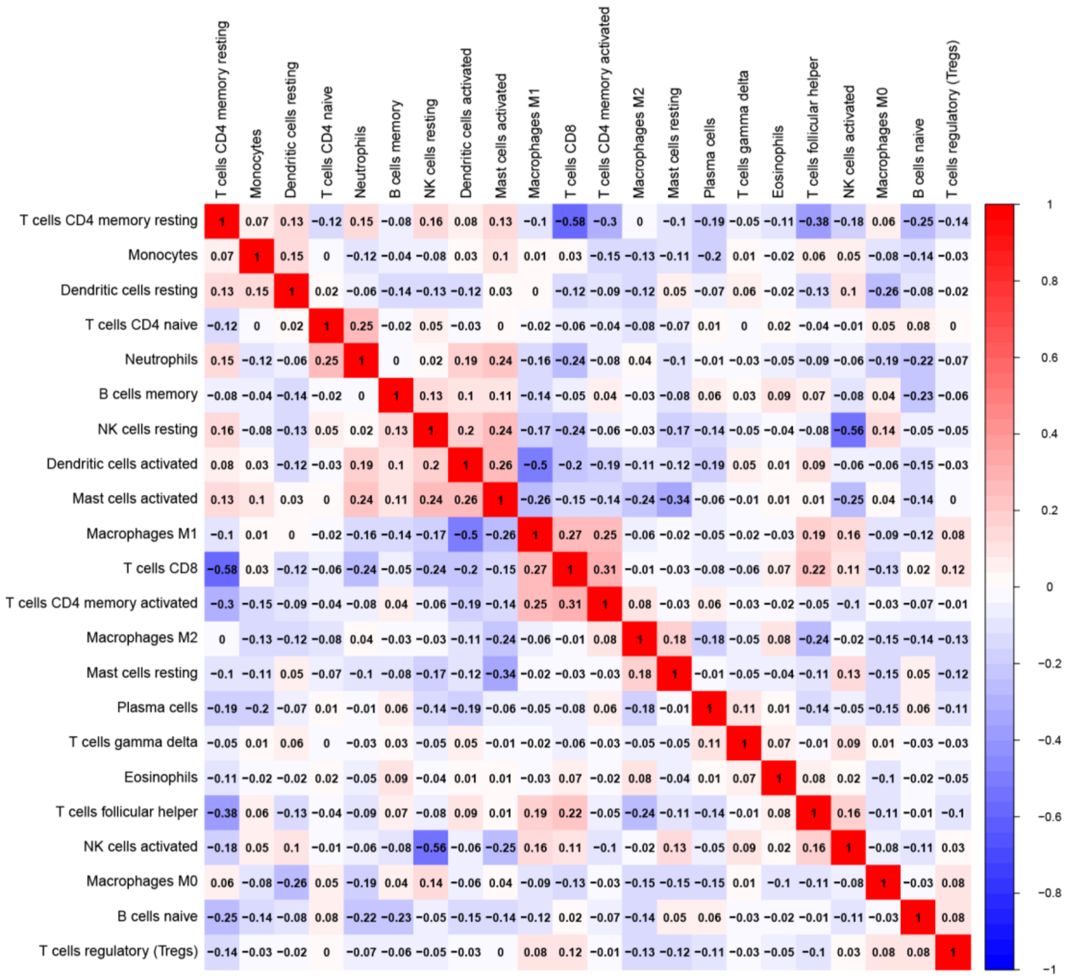

B

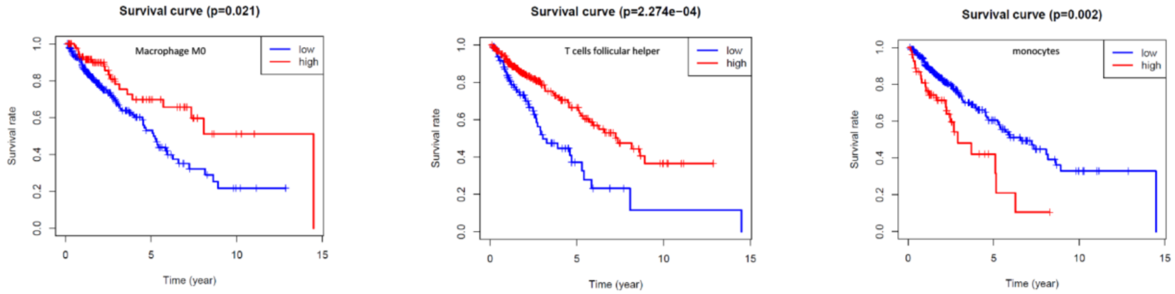

Supplementary Figure 4. (**A**) The heat map shows the proportions of 22 immune cell types in the TCGA-LUSC tumor samples as calculated with the CIBERSORT algorithm, and their correlation with the expression levels of the four hub genes, namely, *LAPTM5, CSF1R, SLCO2B1* and *C1QC* in the TCGA-LUSC dataset. (**B**) The correlation analysis between the proportions of follicular helper T cells, M0 macrophages and monocytes in the LUSC tissues and the overall survival time of the TCGA-LUSC patients. The red and blue lines indicate the LUSC samples with high or low proportions of follicular helper T cells, M0 macrophages, and monocytes.
