# Supplementary material for: Four hub genes regulate tumor infiltration by immune cells, antitumor immunity in the tumor microenvironment, and survival outcomes in lung squamous cell carcinoma patients
Source: Aging (Albany NY). 2021 Jan 10;13(3):3819–42. doi: 10.18632/aging.202351 (PMC7906216; doi:10.18632/aging.202351)
Supplement: Supplementary Table 1 [file aging-13-202351-s002.pdf]

SUPPLEMENTARY TABLE

Supplementary Table 1. Direct website links to the protein expression data of the 4 hub genes in normal lung and LUSC tissues in the human protein atlas database.

Hub genes	Normal lung tissue	LUSC tissue
LAPTM5	https://www.proteinatlas.org/ENSG00000162511-LAPTM5/tissue/bronchus#img	https://www.proteinatlas.org/ENSG00000162511-LAPTM5/pathology/lung+cancer#img
SLCO2B1	https://www.proteinatlas.org/ENSG00000137491-SLCO2B1/tissue/bronchus#img	https://www.proteinatlas.org/ENSG00000137491-SLCO2B1/pathology/lung+cancer#img
C1QC	https://www.proteinatlas.org/ENSG00000159189-C1QC/tissue/bronchus#img	https://www.proteinatlas.org/ENSG00000159189-C1QC/pathology/lung+cancer#img
CSF1R	https://www.proteinatlas.org/ENSG00000182578-CSF1R/tissue/bronchus#img	https://www.proteinatlas.org/ENSG00000182578-CSF1R/pathology/lung+cancer#img
