# Supplementary material for: Four hub genes regulate tumor infiltration by immune cells, antitumor immunity in the tumor microenvironment, and survival outcomes in lung squamous cell carcinoma patients
Source: Aging (Albany NY). 2021 Jan 10;13(3):3819–42. doi: 10.18632/aging.202351 (PMC7906216; doi:10.18632/aging.202351)
Supplement: Supplementary File 1 [file aging-13-202351-s003.docx]

Supplementary File 1. Characteristics of the included TCGA-LUSC patients.

|  | TCGA id | Vital status | Days to_  death | Days to last_  Follow-up | gender | age | M grade | N grade | T grade | stage |
| --- | --- | --- | --- | --- | --- | --- | --- | --- | --- | --- |
|  | tcga-xc-aa0x | dead | NA | NA | female | 77 | m0 | n0 | t1a | 1 |
|  | tcga-o2-a5ib | dead | NA | NA | female | 71 | mx | n1 | t3 | 3 |
|  | tcga-o2-a52w | dead | NA | NA | male | 63 | mx | n0 | t2 | 1 |
|  | tcga-o2-a52v | dead | NA | NA | female | 75 | mx | n0 | t3 | 2 |
|  | tcga-o2-a52s | dead | NA | NA | female | 57 | mx | n2 | t4 | 3 |
|  | tcga-o2-a52q | dead | NA | NA | female | 44 | mx | n1 | t3 | 3 |
|  | tcga-o2-a52n | dead | NA | NA | male | 78 | mx | n0 | t2 | 1 |
|  | tcga-nk-a7xe | alive | NA | 13 | male | 66 | m0 | n2 | t4 | 3 |
|  | tcga-nk-a5d1 | alive | NA | 511 | male | 57 | m0 | n1 | t2a | 2 |
|  | tcga-nk-a5cx | alive | NA | 111 | male | 73 | mx | n0 | t2b | 2 |
|  | tcga-nk-a5ct | alive | NA | 1992 | male | 70 | m0 | n0 | t1 | 1 |
|  | tcga-nk-a5cr | alive | NA | 2542 | male | 77 | mx | n0 | t2 | 1 |
|  | tcga-nc-a5ht | alive | NA | 693 | male | 69 | m0 | n1 | t3 | 3 |
|  | tcga-nc-a5hr | alive | NA | 905 | female | 75 | m0 | n1 | t2a | 2 |
|  | tcga-nc-a5hq | dead | NA | NA | male | 70 | m0 | n2 | t3 | 3 |
|  | tcga-nc-a5hp | alive | NA | 730 | male | 69 | m1b | n0 | t2a | 4 |
|  | tcga-nc-a5ho | alive | NA | 930 | female | 70 | m0 | n1 | t3 | 3 |
|  | tcga-nc-a5hn | alive | NA | 1132 | male | 77 | m0 | n1 | t2a | 2 |
|  | tcga-nc-a5hm | alive | NA | 1212 | male | 76 | m0 | n0 | t2a | 1 |
|  | tcga-nc-a5hl | dead | NA | NA | male | 73 | m0 | n0 | t2b | 2 |
|  | tcga-nc-a5hk | alive | NA | 128 | female | 58 | m0 | n0 | t3 | 2 |
|  | tcga-nc-a5hj | dead | NA | NA | male | 59 | m0 | n0 | t3 | 2 |
|  | tcga-nc-a5hi | alive | NA | 1380 | female | 68 | m0 | n0 | t2 | 1 |
|  | tcga-nc-a5hh | alive | NA | 37 | male | 53 | m0 | n0 | t1 | 1 |
|  | tcga-nc-a5hg | alive | NA | 1575 | male | 59 | m0 | n2 | t2 | 3 |
|  | tcga-nc-a5hf | dead | NA | NA | male | 74 | mx | n0 | t4 | 3 |
|  | tcga-nc-a5he | alive | NA | 1949 | male | 60 | m0 | n1 | t2 | 2 |
|  | tcga-nc-a5hd | dead | NA | NA | male | 79 | m0 | n0 | t3 | 2 |
|  | tcga-mf-a522 | alive | 360 | NA | male | 54 | mx | n0 | t2a | 1 |
|  | tcga-la-a7sw | alive | 408 | NA | male | 71 | mx | n1 | t3 | 3 |
|  | tcga-la-a446 | alive | NA | 401 | male | 68 | mx | n0 | t1b | 1 |
|  | tcga-l3-a524 | alive | 490 | NA | female | 45 | m0 | n0 | t3 | 2 |
|  | tcga-l3-a4e7 | alive | NA | 392 | male | 71 | m0 | n0 | t2a | 1 |
|  | tcga-j1-a4ah | alive | NA | 581 | male | 70 | mx | n0 | t2b | 2 |
|  | tcga-98-a53j | alive | NA | 630 | male | 77 | m0 | n0 | t2a | 1 |
|  | tcga-98-a53i | alive | NA | 565 | male | 64 | m0 | n1 | t2a | 2 |
|  | tcga-98-a53h | alive | NA | 618 | female | 76 | m0 | n0 | t1a | 1 |
|  | tcga-98-a53d | alive | 645 | NA | male | 68 | m0 | n0 | t3 | 2 |
|  | tcga-98-a53c | alive | NA | 822 | female | 77 | m0 | n0 | t1a | 1 |
|  | tcga-98-a53b | dead | NA | NA | male | 69 | m0 | n0 | t2a | 1 |
|  | tcga-98-a53a | alive | 552 | NA | male | 70 | m0 | n0 | t2a | 1 |
|  | tcga-98-a539 | alive | NA | 173 | male | 63 | m0 | n0 | t3 | 2 |
|  | tcga-98-a538 | alive | NA | 826 | male | 67 | m0 | n0 | t3 | 2 |
|  | tcga-98-8023 | alive | NA | 649 | male | 70 | m0 | n1 | t3 | 3 |
|  | tcga-98-8022 | alive | 933 | NA | male | 61 | m0 | n0 | t1a | 1 |
|  | tcga-98-8021 | alive | NA | 937 | female | 75 | m0 | n0 | t1a | 1 |
|  | tcga-98-8020 | dead | NA | NA | female | 56 | m0 | n2 | t2 | 3 |
|  | tcga-98-7454 | alive | NA | 256 | male | 73 | m0 | n0 | t2a | 1 |
|  | tcga-96-a4jl | alive | NA | 504 | female | 78 | m0 | n1 | t2a | 2 |
|  | tcga-96-a4jk | alive | NA | 589 | male | 65 | m0 | n1 | t2a | 2 |
|  | tcga-96-8170 | alive | NA | 531 | female | 75 | m0 | n1 | t1a | 2 |
|  | tcga-96-8169 | alive | NA | 557 | female | 67 | m0 | n0 | t1a | 1 |
|  | tcga-96-7545 | alive | NA | 1344 | male | 73 | mx | n0 | t1 | 1 |
|  | tcga-96-7544 | alive | NA | 1517 | male | 83 | mx | n1 | t2 | 2 |
|  | tcga-94-a5i6 | alive | NA | 538 | male | 62 | m0 | n0 | t3 | 2 |
|  | tcga-94-a5i4 | alive | NA | 491 | male | 61 | mx | n1 | t2a | 2 |
|  | tcga-94-a4vj | alive | NA | 430 | female | 71 | m0 | n0 | t1b | 1 |
|  | tcga-94-8491 | alive | NA | 810 | male | 73 | m0 | n1 | t2a | 2 |
|  | tcga-94-8490 | alive | NA | 153 | male | 70 | m0 | n0 | t3 | 2 |
|  | tcga-94-8035 | alive | NA | 122 | male | 64 | mx | n0 | t3 | 2 |
|  | tcga-94-7943 | alive | NA | 143 | male | 80 | mx | nx | t1b | 1 |
|  | tcga-94-7557 | dead | 5 | NA | male | 73 | m0 | n0 | t2 | 1 |
|  | tcga-94-7033 | alive | NA | 87 | male | 73 | mx | n0 | t2 | 1 |
|  | tcga-92-8065 | alive | NA | 70 | female | 74 | mx | n0 | t3 | 2 |
|  | tcga-92-8064 | alive | NA | 160 | male | 58 | mx | n0 | t2b | 1 |
|  | tcga-92-8063 | alive | NA | 122 | male | 52 | mx | n2 | t2b | 3 |
|  | tcga-92-7341 | alive | NA | 106 | male | 71 | mx | n0 | t2a | 1 |
|  | tcga-92-7340 | alive | NA | 82 | female | 45 | mx | n1 | t2a | 2 |
|  | tcga-90-a59q | alive | 322 | NA | female | 61 | mx | n1 | t2a | 2 |
|  | tcga-90-a4ee | alive | NA | 688 | male | 53 | mx | n1 | t2a | 2 |
|  | tcga-90-a4ed | alive | NA | 615 | male | 69 | mx | n0 | t2a | 1 |
|  | tcga-90-7964 | alive | NA | 428 | male | 70 | mx | n0 | t2a | 1 |
|  | tcga-90-7769 | alive | NA | 358 | male | 55 | mx | n1 | t2b | 2 |
|  | tcga-90-7767 | alive | NA | 89 | male | 56 | mx | n1 | t2b | 2 |
|  | tcga-90-7766 | alive | NA | 289 | female | 66 | mx | n0 | t1b | 1 |
|  | tcga-90-6837 | alive | NA | 758 | male | 64 | mx | n0 | t3 | 2 |
|  | tcga-85-a5b5 | alive | NA | NA | male | 58 | m0 | n0 | t1b | 1 |
|  | tcga-85-a53l | alive | NA | 377 | male | 63 | m0 | n0 | t2b | 2 |
|  | tcga-85-a513 | alive | NA | 461 | female | 60 | m0 | nx | t1a | 1 |
|  | tcga-85-a512 | alive | NA | 465 | male | 46 | m0 | n1 | t1b | 2 |
|  | tcga-85-a511 | alive | 455 | NA | male | 62 | m0 | n1 | t2b | 2 |
|  | tcga-85-a510 | alive | NA | 482 | female | 74 | m0 | n1 | t2b | 2 |
|  | tcga-85-a50z | alive | NA | 493 | male | 57 | m0 | n0 | t2b | 2 |
|  | tcga-85-a50m | alive | NA | 382 | male | 47 | m0 | n0 | t2b | 2 |
|  | tcga-85-a4qr | alive | NA | 600 | male | 67 | m0 | n0 | t2a | 1 |
|  | tcga-85-a4qq | alive | NA | 553 | male | 68 | m0 | n0 | t2a | 1 |
|  | tcga-85-a4pa | alive | NA | 741 | male | 61 | m0 | n0 | t2a | 1 |
|  | tcga-85-a4jc | alive | NA | 455 | male | 84 | m0 | n0 | t2b | 2 |
|  | tcga-85-a4jb | alive | NA | 539 | male | 74 | m0 | n0 | t3 | 2 |
|  | tcga-85-a4cn | alive | NA | 612 | female | 56 | m0 | n1 | t2b | 2 |
|  | tcga-85-a4cl | alive | NA | 781 | male | 65 | m0 | n0 | t1b | 1 |
|  | tcga-85-8666 | alive | NA | 475 | male | 65 | m0 | n0 | t2a | 1 |
|  | tcga-85-8664 | alive | NA | 434 | male | 73 | m0 | n1 | t2b | 2 |
|  | tcga-85-8584 | alive | 383 | NA | male | 71 | m0 | n1 | t2a | 2 |
|  | tcga-85-8582 | alive | NA | 424 | male | 49 | m0 | n0 | t1a | 1 |
|  | tcga-85-8580 | alive | NA | 445 | female | 52 | m0 | n0 | t2a | 1 |
|  | tcga-85-8481 | alive | 236 | NA | male | 70 | m0 | n0 | t3 | 2 |
|  | tcga-85-8479 | alive | NA | 468 | male | 66 | m0 | n0 | t1a | 1 |
|  | tcga-85-8355 | alive | NA | 61 | male | 63 | m0 | n0 | t1a | 1 |
|  | tcga-85-8354 | alive | NA | 658 | male | 53 | m0 | n0 | t2a | 1 |
|  | tcga-85-8353 | alive | 94 | NA | male | 72 | m0 | n1 | t3 | 3 |
|  | tcga-85-8352 | alive | 161 | NA | male | 67 | m0 | n1 | t3 | 3 |
|  | tcga-85-8351 | alive | NA | 510 | male | 72 | m0 | n1 | t2a | 2 |
|  | tcga-85-8350 | alive | NA | 683 | male | 61 | m0 | n0 | t1b | 1 |
|  | tcga-85-8288 | alive | 402 | NA | male | 70 | m0 | n1 | t1b | 2 |
|  | tcga-85-8287 | alive | 23 | NA | male | 72 | m0 | n0 | t1b | 1 |
|  | tcga-85-8277 | alive | 307 | NA | male | 70 | m0 | n1 | t3 | 3 |
|  | tcga-85-8276 | alive | NA | 1050 | male | 62 | m0 | n1 | t1b | 2 |
|  | tcga-85-8072 | alive | NA | 513 | male | 60 | m0 | n0 | t1a | 1 |
|  | tcga-85-8071 | alive | NA | 428 | male | 52 | m0 | n1 | t1a | 2 |
|  | tcga-85-8070 | alive | NA | 510 | male | 71 | m0 | n0 | t2 | 1 |
|  | tcga-85-8052 | alive | NA | 734 | male | 53 | m0 | n0 | t3 | 2 |
|  | tcga-85-8049 | alive | NA | 579 | male | 57 | m0 | n0 | t2a | 1 |
|  | tcga-85-8048 | alive | NA | 765 | male | 62 | m0 | n0 | t1 | 1 |
|  | tcga-85-7950 | alive | NA | 576 | male | 46 | m0 | n0 | t2a | 1 |
|  | tcga-85-7844 | alive | NA | 911 | male | 71 | m0 | n0 | t2a | 1 |
|  | tcga-85-7843 | alive | NA | NA | male | 50 | m0 | n1 | t2a | 2 |
|  | tcga-85-7710 | alive | NA | 42 | female | 59 | m0 | n0 | t1b | 1 |
|  | tcga-85-7699 | alive | 1001 | NA | male | 73 | m0 | n0 | t4 | 3 |
|  | tcga-85-7698 | alive | NA | 952 | male | 48 | m0 | n0 | t1b | 1 |
|  | tcga-85-7697 | alive | NA | 1063 | male | 49 | m0 | n0 | t3 | 2 |
|  | tcga-85-7696 | alive | NA | 376 | male | 64 | m0 | n0 | t1 | 1 |
|  | tcga-85-6798 | alive | 195 | NA | male | 57 | m0 | n1 | t3 | 3 |
|  | tcga-85-6561 | alive | NA | 395 | male | 66 | m0 | nx | t2a | 1 |
|  | tcga-85-6560 | alive | NA | 364 | male | 59 | m0 | n1 | t1b | 2 |
|  | tcga-85-6175 | alive | 294 | NA | female | 63 | m0 | n0 | t3 | 2 |
|  | tcga-79-5596 | alive | NA | NA | male | NA | m0 | n1 | t3 | 3 |
|  | tcga-77-a5gh | alive | NA | 713 | male | 81 | m0 | n0 | t2a | 1 |
|  | tcga-77-a5gf | alive | NA | 839 | male | 70 | m0 | n1 | t2a | 2 |
|  | tcga-77-a5gb | dead | NA | NA | male | 90 | m0 | nx | t2a | 1 |
|  | tcga-77-a5ga | alive | NA | 1280 | male | 76 | m0 | n0 | t2a | 1 |
|  | tcga-77-a5g8 | alive | NA | 1290 | male | 70 | m0 | n0 | t3 | 2 |
|  | tcga-77-a5g7 | dead | NA | NA | male | 63 | m0 | n0 | t1 | 1 |
|  | tcga-77-a5g6 | dead | NA | NA | male | 66 | m0 | n2 | t2 | 3 |
|  | tcga-77-a5g3 | alive | NA | 3576 | male | 63 | m0 | n1 | t2 | 2 |
|  | tcga-77-a5g1 | alive | NA | 4026 | male | 75 | m0 | n1 | t3 | 3 |
|  | tcga-77-a5fz | dead | NA | NA | male | 64 | m0 | n0 | t4 | 3 |
|  | tcga-77-8156 | alive | NA | 10 | male | 60 | m0 | n0 | t2a | 1 |
|  | tcga-77-8154 | alive | NA | 1092 | male | 67 | m0 | n0 | t1 | 1 |
|  | tcga-77-8153 | alive | NA | 1992 | female | 77 | m0 | n0 | t2 | 1 |
|  | tcga-77-8150 | dead | 1655 | NA | male | 64 | m0 | n1 | t3 | 3 |
|  | tcga-77-8148 | alive | NA | 293 | male | 68 | m0 | n1 | t3 | 3 |
|  | tcga-77-8146 | alive | NA | 2183 | male | 72 | m0 | n0 | t1 | 1 |
|  | tcga-77-8145 | dead | 212 | NA | male | 73 | m0 | n1 | t4 | 3 |
|  | tcga-77-8144 | alive | NA | 833 | male | 70 | m0 | n0 | t2 | 1 |
|  | tcga-77-8143 | dead | 803 | NA | male | 76 | m0 | n2 | t2 | 3 |
|  | tcga-77-8140 | dead | 351 | NA | female | 66 | m0 | n1 | t2 | 2 |
|  | tcga-77-8139 | alive | NA | 3166 | male | 72 | m0 | n0 | t3 | 2 |
|  | tcga-77-8138 | dead | 539 | NA | male | 74 | m0 | n0 | t2 | 1 |
|  | tcga-77-8136 | dead | 1189 | NA | female | 74 | m0 | n1 | t2 | 2 |
|  | tcga-77-8133 | dead | 1640 | NA | male | 74 | m0 | n1 | t1 | 2 |
|  | tcga-77-8131 | dead | 383 | NA | male | 72 | m0 | nx | t2 | 1 |
|  | tcga-77-8130 | alive | NA | 2522 | male | 69 | m0 | n1 | t2 | 2 |
|  | tcga-77-8128 | dead | 1150 | NA | male | 60 | m0 | n2 | t2 | 3 |
|  | tcga-77-8009 | alive | NA | 1641 | male | 68 | m0 | n1 | t2 | 2 |
|  | tcga-77-8008 | dead | 2639 | NA | male | 68 | m0 | n0 | t2 | 1 |
|  | tcga-77-8007 | dead | 198 | NA | male | 68 | m0 | n1 | t2 | 2 |
|  | tcga-77-7465 | alive | NA | 479 | male | 58 | m0 | n1 | t2a | 2 |
|  | tcga-77-7463 | dead | 1423 | NA | male | 75 | m0 | n0 | t2 | 1 |
|  | tcga-77-7338 | dead | 5 | NA | male | 64 | m0 | n0 | t2 | 1 |
|  | tcga-77-7337 | dead | 3253 | NA | male | 65 | m0 | n1 | t2 | 2 |
|  | tcga-77-7335 | dead | 2133 | NA | female | 62 | m0 | n2 | t4 | 3 |
|  | tcga-77-7142 | alive | NA | 1286 | female | 59 | m0 | n0 | t2 | 1 |
|  | tcga-77-7141 | alive | NA | 15 | male | 64 | m0 | n0 | t2 | 1 |
|  | tcga-77-7140 | dead | 632 | NA | female | 69 | m0 | n1 | t2 | 2 |
|  | tcga-77-7139 | alive | NA | 2737 | male | 56 | m0 | n1 | t2 | 2 |
|  | tcga-77-7138 | dead | 340 | NA | male | 67 | m0 | n0 | t2 | 1 |
|  | tcga-77-6845 | dead | 708 | NA | male | 69 | m0 | n0 | t3 | 2 |
|  | tcga-77-6844 | dead | 2284 | NA | male | 74 | m0 | n1 | t3 | 3 |
|  | tcga-77-6843 | dead | 2224 | NA | male | 74 | m0 | n1 | t1 | 2 |
|  | tcga-77-6842 | dead | 899 | NA | male | 79 | m0 | n1 | t2 | 2 |
|  | tcga-70-6723 | alive | NA | 375 | male | 65 | m0 | n0 | t3 | 2 |
|  | tcga-70-6722 | alive | NA | 367 | male | 47 | m0 | n1 | t3 | 3 |
|  | tcga-6a-ab49 | dead | NA | NA | female | 73 | mx | n0 | t2 | 1 |
|  | tcga-68-a59j | alive | NA | 448 | female | 74 | mx | n0 | t2a | 1 |
|  | tcga-68-a59i | alive | NA | 492 | female | 73 | m0 | n1 | t3 | 3 |
|  | tcga-68-8251 | alive | NA | 406 | male | 78 | m0 | n0 | t2a | 1 |
|  | tcga-68-8250 | alive | NA | 244 | male | 66 | mx | n0 | t1a | 1 |
|  | tcga-68-7757 | alive | NA | 211 | male | 74 | mx | n0 | t1b | 1 |
|  | tcga-68-7756 | alive | NA | 202 | male | 84 | mx | n1 | t4 | 3 |
|  | tcga-68-7755 | alive | NA | 83 | female | 60 | m0 | n1 | t1b | 2 |
|  | tcga-66-2800 | alive | NA | 1492 | male | 70 | m0 | n0 | t4 | 3 |
|  | tcga-66-2795 | alive | NA | NA | male | 68 | m0 | n1 | t4 | 3 |
|  | tcga-66-2794 | alive | NA | 1645 | male | 64 | m0 | n2 | t4 | 3 |
|  | tcga-66-2793 | dead | NA | NA | male | 68 | m0 | n1 | t4 | 3 |
|  | tcga-66-2792 | alive | NA | 913 | male | 58 | m0 | n1 | t2 | 2 |
|  | tcga-66-2791 | dead | NA | NA | male | 66 | m0 | n3 | t2 | 3 |
|  | tcga-66-2790 | alive | NA | 699 | male | 72 | m0 | n1 | t2 | 2 |
|  | tcga-66-2789 | dead | NA | NA | male | 73 | m0 | n3 | t1 | 3 |
|  | tcga-66-2788 | alive | NA | 699 | male | 56 | m0 | n0 | t2 | 1 |
|  | tcga-66-2787 | alive | NA | 1217 | male | 57 | m0 | n0 | t1 | 1 |
|  | tcga-66-2786 | alive | NA | 790 | female | 68 | m0 | n0 | t1 | 1 |
|  | tcga-66-2785 | alive | NA | NA | male | 65 | m0 | n0 | t2 | 1 |
|  | tcga-66-2783 | alive | NA | 759 | male | 67 | m0 | n3 | t2 | 3 |
|  | tcga-66-2782 | dead | 365 | NA | male | 71 | m0 | n0 | t3 | 2 |
|  | tcga-66-2781 | alive | NA | NA | male | 67 | m0 | n0 | t2 | 1 |
|  | tcga-66-2780 | dead | NA | NA | male | 65 | m0 | n0 | t2 | 1 |
|  | tcga-66-2778 | alive | NA | 578 | female | 68 | m0 | n3 | t2 | 3 |
|  | tcga-66-2777 | alive | NA | NA | male | 71 | m0 | n0 | t2 | 1 |
|  | tcga-66-2773 | dead | NA | NA | male | 69 | m0 | n0 | t2 | 1 |
|  | tcga-66-2771 | alive | NA | 578 | male | 60 | m0 | n1 | t2 | 2 |
|  | tcga-66-2770 | alive | NA | NA | male | 79 | m0 | n0 | t2 | 1 |
|  | tcga-66-2769 | dead | NA | NA | male | 75 | m0 | n0 | t4 | 3 |
|  | tcga-66-2768 | alive | NA | NA | male | 57 | m0 | n1 | t2 | 2 |
|  | tcga-66-2767 | alive | NA | NA | male | 62 | m0 | n3 | t2 | 3 |
|  | tcga-66-2766 | alive | NA | NA | male | 54 | m0 | n2 | t2 | 3 |
|  | tcga-66-2765 | alive | NA | NA | male | 64 | m0 | n0 | t2 | 1 |
|  | tcga-66-2763 | alive | NA | NA | female | 63 | m0 | n0 | t2 | 1 |
|  | tcga-66-2759 | alive | NA | 762 | male | 66 | m0 | n2 | t2 | 3 |
|  | tcga-66-2758 | alive | NA | 639 | male | 71 | m0 | n0 | t2 | 1 |
|  | tcga-66-2757 | alive | 1338 | NA | female | 65 | m0 | n0 | t1 | 1 |
|  | tcga-66-2756 | alive | NA | NA | male | 68 | m0 | n0 | t4 | 3 |
|  | tcga-66-2755 | alive | NA | NA | male | 63 | m0 | n0 | t2 | 1 |
|  | tcga-66-2754 | alive | NA | NA | male | 67 | m0 | n2 | t2 | 3 |
|  | tcga-66-2753 | alive | NA | NA | male | 69 | m0 | n0 | t2 | 1 |
|  | tcga-66-2744 | alive | NA | NA | male | 71 | m0 | n1 | t2 | 2 |
|  | tcga-66-2742 | alive | NA | 641 | male | 70 | m1 | n1 | t2 | 4 |
|  | tcga-66-2737 | alive | NA | NA | male | 72 | m0 | n1 | t2 | 2 |
|  | tcga-66-2734 | alive | NA | 1311 | female | 62 | m0 | n0 | t2 | 1 |
|  | tcga-66-2727 | dead | NA | NA | female | 55 | m0 | n0 | t2 | 1 |
|  | tcga-63-a5my | alive | NA | 1052 | male | 63 | m0 | n0 | t1b | 1 |
|  | tcga-63-a5mw | alive | NA | 1639 | male | 76 | m0 | n0 | t2 | 1 |
|  | tcga-63-a5mv | alive | NA | 1100 | male | 69 | m0 | n0 | t2b | 2 |
|  | tcga-63-a5mu | dead | 773 | NA | male | 48 | m0 | n1 | t2b | 2 |
|  | tcga-63-a5mt | alive | NA | NA | male | 74 | m0 | n0 | t3 | 2 |
|  | tcga-63-a5ms | alive | NA | 2381 | male | 78 | m0 | n0 | t2 | 1 |
|  | tcga-63-a5mr | alive | NA | 2716 | female | 70 | m0 | n0 | t2 | 1 |
|  | tcga-63-a5mp | alive | NA | NA | male | 56 | m0 | n1 | t2 | 2 |
|  | tcga-63-a5mn | dead | 345 | NA | female | 78 | m0 | n0 | t3 | 2 |
|  | tcga-63-a5mm | dead | 456 | NA | female | 69 | m0 | n1 | t2 | 2 |
|  | tcga-63-a5ml | alive | NA | 1386 | male | 68 | m0 | n0 | t2 | 1 |
|  | tcga-63-a5mj | alive | NA | 1824 | male | 54 | m0 | n1 | t2 | 2 |
|  | tcga-63-a5mi | alive | NA | 1784 | male | 65 | m0 | n2 | t2 | 3 |
|  | tcga-63-a5mh | alive | NA | 2026 | male | 68 | m0 | n0 | t1 | 1 |
|  | tcga-63-a5mg | alive | NA | 2148 | male | 68 | m0 | n0 | t2 | 1 |
|  | tcga-63-a5mb | alive | NA | 3123 | male | 62 | m0 | n0 | t2 | 1 |
|  | tcga-63-a5m9 | alive | NA | 0 | female | NA | m0 | n1 | t2 | 2 |
|  | tcga-63-7023 | alive | NA | NA | male | NA | m0 | n1 | t1 | 2 |
|  | tcga-63-7022 | alive | NA | NA | female | NA | m0 | n0 | t1 | 1 |
|  | tcga-63-7021 | alive | NA | NA | male | NA | m0 | n0 | t1 | 1 |
|  | tcga-63-7020 | alive | NA | NA | male | NA | m0 | n0 | t1 | 1 |
|  | tcga-63-6202 | alive | NA | NA | male | NA | m0 | n0 | t2 | 2 |
|  | tcga-63-5131 | dead | NA | NA | male | NA | m0 | n1 | t2 | 2 |
|  | tcga-63-5128 | dead | NA | NA | male | NA | m0 | n0 | t2 | 1 |
|  | tcga-60-2726 | dead | 358 | 358 | male | 56 | m0 | n1 | t2 | 2 |
|  | tcga-60-2725 | alive | NA | 816 | male | 74 | m0 | n0 | t2 | 1 |
|  | tcga-60-2724 | alive | NA | 717 | male | 47 | m0 | n1 | t3 | 3 |
|  | tcga-60-2723 | alive | NA | 693 | female | 74 | m0 | n0 | t2 | 1 |
|  | tcga-60-2722 | alive | NA | 908 | male | 66 | m0 | n1 | t2 | 2 |
|  | tcga-60-2721 | alive | NA | 983 | male | 73 | m0 | n0 | t2 | 1 |
|  | tcga-60-2720 | alive | NA | 97 | female | 60 | m0 | n0 | t2 | 1 |
|  | tcga-60-2719 | alive | NA | 932 | female | 83 | m0 | n0 | t1 | 1 |
|  | tcga-60-2716 | alive | NA | 1356 | male | 39 | m0 | n1 | t2 | 2 |
|  | tcga-60-2715 | dead | 1075 | NA | male | 51 | m0 | n0 | t1 | 1 |
|  | tcga-60-2714 | alive | NA | 512 | female | 66 | m0 | n1 | t2 | 2 |
|  | tcga-60-2713 | alive | NA | 1580 | male | 64 | m0 | n0 | t2 | 1 |
|  | tcga-60-2712 | dead | 274 | 274 | female | 79 | m0 | n1 | t2 | 2 |
|  | tcga-60-2711 | alive | NA | 1014 | female | 64 | m0 | n0 | t2 | 1 |
|  | tcga-60-2710 | alive | NA | 1714 | female | 67 | m0 | n1 | t1 | 2 |
|  | tcga-60-2709 | alive | NA | 1505 | male | 69 | mx | n0 | t2 | 1 |
|  | tcga-60-2708 | alive | NA | 2447 | female | 64 | m0 | n1 | t2 | 2 |
|  | tcga-60-2707 | dead | NA | 661 | male | 70 | m0 | n0 | t2 | 1 |
|  | tcga-60-2706 | alive | NA | 2500 | male | 58 | m0 | n0 | t1 | 1 |
|  | tcga-60-2704 | dead | 1154 | NA | male | 73 | m0 | n1 | t2 | 2 |
|  | tcga-60-2703 | dead | 2945 | NA | male | 73 | m0 | n1 | t2 | 2 |
|  | tcga-60-2698 | dead | 311 | NA | male | 62 | m0 | n1 | t2 | 2 |
|  | tcga-60-2697 | dead | 372 | NA | male | 41 | m0 | n2 | t2 | 2 |
|  | tcga-60-2696 | dead | 109 | 109 | female | 76 | m0 | n0 | t2 | 2 |
|  | tcga-60-2695 | alive | NA | 642 | female | 74 | m0 | n0 | t2 | 1 |
|  | tcga-58-a46n | alive | NA | 910 | male | 52 | m0 | n0 | t2a | 1 |
|  | tcga-58-a46m | alive | NA | 1072 | male | 61 | m0 | n1 | t2b | 2 |
|  | tcga-58-a46l | alive | NA | 1723 | male | 73 | m0 | n2 | t2 | 3 |
|  | tcga-58-a46k | dead | NA | NA | male | 59 | m0 | n2 | t2 | 3 |
|  | tcga-58-a46j | alive | NA | 2589 | male | 64 | m0 | n1 | t2 | 2 |
|  | tcga-58-8393 | alive | NA | 1058 | female | 68 | m0 | n0 | t2a | 1 |
|  | tcga-58-8392 | dead | NA | NA | male | 70 | m0 | n0 | t2a | 1 |
|  | tcga-58-8391 | alive | NA | 2167 | female | 57 | m0 | n2 | t2 | 3 |
|  | tcga-58-8390 | alive | NA | 911 | male | 70 | m0 | n0 | t2b | 2 |
|  | tcga-58-8388 | alive | 412 | NA | male | 60 | m0 | n0 | t2a | 1 |
|  | tcga-58-8387 | dead | NA | NA | male | 60 | m0 | n0 | t2b | 2 |
|  | tcga-58-8386 | dead | NA | NA | male | 75 | m1 | nx | t3 | 4 |
|  | tcga-56-a62t | alive | NA | 84 | male | 78 | mx | n0 | t2b | 2 |
|  | tcga-56-a5ds | alive | NA | 8 | female | 72 | mx | n0 | t2a | 1 |
|  | tcga-56-a5dr | alive | NA | 4 | male | 81 | mx | n0 | t1a | 1 |
|  | tcga-56-a4zk | alive | NA | 570 | female | 76 | m0 | n0 | t2 | 1 |
|  | tcga-56-a4zj | alive | NA | 640 | female | 75 | m0 | n0 | t1a | 1 |
|  | tcga-56-a4by | alive | 543 | NA | male | 66 | mx | n0 | t2a | 1 |
|  | tcga-56-a4bx | alive | NA | 405 | male | 70 | mx | n0 | t2b | 2 |
|  | tcga-56-a4bw | alive | NA | 585 | male | 55 | m0 | n1 | t2a | 2 |
|  | tcga-56-a49d | alive | NA | 637 | male | 67 | mx | n2 | t2a | 3 |
|  | tcga-56-8629 | alive | NA | 481 | male | 63 | mx | n0 | t2b | 2 |
|  | tcga-56-8628 | alive | NA | 616 | male | 78 | mx | n0 | t1b | 1 |
|  | tcga-56-8626 | alive | 302 | NA | male | 59 | mx | n0 | t1a | 1 |
|  | tcga-56-8625 | alive | 315 | NA | female | 66 | mx | n1 | t3 | 3 |
|  | tcga-56-8624 | alive | NA | 420 | male | 84 | mx | n0 | t3 | 2 |
|  | tcga-56-8623 | alive | 692 | NA | male | 71 | mx | n0 | t2a | 1 |
|  | tcga-56-8622 | alive | NA | NA | male | 68 | m0 | n0 | t2a | 1 |
|  | tcga-56-8504 | alive | NA | 510 | male | 74 | mx | n0 | t2a | 1 |
|  | tcga-56-8503 | alive | NA | NA | female | 76 | m0 | n0 | t3 | 2 |
|  | tcga-56-8309 | alive | NA | 428 | male | 66 | mx | n0 | t1b | 1 |
|  | tcga-56-8308 | alive | NA | 517 | male | 79 | mx | n0 | t3 | 2 |
|  | tcga-56-8307 | alive | NA | 818 | female | 55 | m0 | n0 | t3 | 2 |
|  | tcga-56-8305 | alive | NA | NA | male | 72 | m0 | n0 | t2a | 1 |
|  | tcga-56-8304 | alive | NA | 106 | female | 73 | mx | n0 | t1b | 1 |
|  | tcga-56-8201 | alive | 397 | NA | male | 74 | mx | n0 | t3 | 2 |
|  | tcga-56-8083 | alive | NA | 150 | male | 56 | mx | n0 | t2a | 1 |
|  | tcga-56-8082 | alive | NA | 455 | female | 80 | mx | n0 | t2b | 2 |
|  | tcga-56-7823 | alive | NA | 1011 | female | 58 | m0 | n1 | t1b | 2 |
|  | tcga-56-7822 | alive | 532 | NA | male | 75 | m0 | n1 | t2b | 2 |
|  | tcga-56-7731 | alive | 3 | NA | female | 66 | mx | n0 | t2a | 1 |
|  | tcga-56-7730 | alive | 198 | NA | male | 73 | m0 | n0 | t2b | 2 |
|  | tcga-56-7582 | alive | NA | 601 | male | 83 | m0 | n0 | t2a | 1 |
|  | tcga-56-7580 | alive | NA | 925 | male | 84 | m0 | n0 | t2a | 1 |
|  | tcga-56-7579 | alive | 951 | NA | male | 61 | m0 | n1 | t3 | 3 |
|  | tcga-56-7223 | alive | 442 | NA | male | 66 | mx | n1 | t3 | 3 |
|  | tcga-56-7222 | alive | 562 | NA | male | 60 | m0 | n0 | t2a | 1 |
|  | tcga-56-7221 | alive | NA | 608 | male | 79 | m0 | n0 | t2 | 1 |
|  | tcga-56-6546 | alive | NA | NA | male | 67 | mx | n0 | t2b | 2 |
|  | tcga-56-6545 | alive | NA | 666 | female | 77 | m0 | n0 | t2a | 1 |
|  | tcga-56-5898 | alive | NA | 555 | male | 69 | m0 | n0 | t1b | 1 |
|  | tcga-56-5897 | alive | NA | 378 | male | 74 | mx | n0 | t1b | 1 |
|  | tcga-56-1622 | dead | 881 | NA | male | 58 | m0 | n0 | t2 | 1 |
|  | tcga-52-7812 | dead | 835 | NA | male | 68 | m0 | n2 | t2 | 2 |
|  | tcga-52-7811 | dead | 266 | NA | male | 67 | m0 | n0 | t2 | 1 |
|  | tcga-52-7810 | alive | NA | 923 | female | 60 | m0 | n0 | t3 | 2 |
|  | tcga-52-7809 | dead | 166 | NA | male | 74 | m0 | n0 | t2 | 1 |
|  | tcga-52-7622 | alive | NA | 862 | female | 62 | m0 | n0 | t1a | 1 |
|  | tcga-51-6867 | dead | 1856 | NA | female | 72 | m0 | n0 | t1 | 1 |
|  | tcga-51-4081 | alive | NA | 63 | male | 55 | m0 | n1 | t2a | 2 |
|  | tcga-51-4080 | dead | 12 | NA | male | 65 | NA | n1 | t4 | 3 |
|  | tcga-51-4079 | dead | 12 | NA | female | 73 | NA | n0 | t2 | 1 |
|  | tcga-46-6026 | alive | NA | 423 | male | 81 | m0 | n1 | t2a | 2 |
|  | tcga-46-6025 | alive | NA | 324 | male | 71 | m0 | n1 | t2b | 2 |
|  | tcga-46-3769 | alive | NA | 135 | male | 57 | m0 | n0 | t4 | 2 |
|  | tcga-46-3768 | dead | 299 | 4 | male | 58 | m0 | n1 | t3 | 3 |
|  | tcga-46-3767 | alive | NA | 388 | male | 76 | m0 | n0 | t1a | 1 |
|  | tcga-46-3766 | alive | NA | -9 | female | 62 | m0 | n0 | t1 | 1 |
|  | tcga-46-3765 | alive | NA | 12 | female | 59 | m0 | n0 | t1 | 1 |
|  | tcga-43-a56v | alive | NA | 366 | male | 61 | m0 | n2 | t2a | 3 |
|  | tcga-43-a56u | alive | NA | 432 | female | 76 | mx | n0 | t1b | 1 |
|  | tcga-43-a475 | alive | NA | 296 | female | 67 | m0 | n0 | t3 | 2 |
|  | tcga-43-a474 | alive | NA | 353 | male | 66 | m0 | n0 | t2b | 2 |
|  | tcga-43-8118 | dead | 89 | NA | female | 55 | m0 | n0 | t1b | 1 |
|  | tcga-43-8116 | alive | NA | 358 | male | 73 | m0 | n0 | t1b | 1 |
|  | tcga-43-8115 | alive | NA | 407 | female | 72 | mx | n1 | t2a | 2 |
|  | tcga-43-7658 | alive | NA | 2023 | female | 75 | m0 | n0 | t1 | 1 |
|  | tcga-43-7657 | alive | NA | 236 | female | 68 | mx | n0 | t1 | 1 |
|  | tcga-43-7656 | alive | NA | 315 | male | 71 | mx | n0 | t1b | 1 |
|  | tcga-43-6773 | dead | 116 | NA | male | 76 | mx | n1 | t2 | 2 |
|  | tcga-43-6771 | dead | 166 | NA | male | 85 | mx | n0 | t2 | 1 |
|  | tcga-43-6770 | alive | NA | 310 | female | 59 | mx | n0 | t2a | 1 |
|  | tcga-43-6647 | alive | NA | 379 | female | 69 | mx | n1 | t2b | 2 |
|  | tcga-43-6143 | alive | NA | 376 | male | 70 | m0 | n0 | t2 | 1 |
|  | tcga-43-5670 | alive | NA | 549 | male | 70 | m0 | n0 | t2b | 2 |
|  | tcga-43-5668 | alive | NA | 349 | male | 78 | m0 | n1 | t1b | 2 |
|  | tcga-43-3920 | alive | NA | 357 | male | 71 | m0 | n0 | t2 | 1 |
|  | tcga-43-3394 | alive | NA | 433 | male | 52 | m0 | n0 | t2a | 1 |
|  | tcga-43-2581 | alive | NA | 399 | female | 47 | m0 | n1 | t3 | 3 |
|  | tcga-43-2578 | alive | NA | 550 | female | 59 | m0 | n0 | t1 | 1 |
|  | tcga-43-2576 | alive | NA | 556 | female | 62 | m0 | n2 | t2 | 3 |
|  | tcga-39-5040 | dead | 519 | NA | male | 59 | m0 | n2 | t2a | 3 |
|  | tcga-39-5039 | dead | 544 | 544 | male | 76 | m0 | n0 | t2b | 2 |
|  | tcga-39-5037 | alive | NA | 1086 | male | 65 | m0 | n1 | t1b | 2 |
|  | tcga-39-5036 | alive | NA | 1084 | male | 73 | m0 | n0 | t2 | 1 |
|  | tcga-39-5035 | alive | NA | 474 | female | 72 | m0 | n0 | t1b | 1 |
|  | tcga-39-5034 | dead | 1107 | 1107 | female | 73 | m0 | n0 | t3 | 2 |
|  | tcga-39-5031 | alive | NA | 833 | female | 76 | m0 | n0 | t1a | 1 |
|  | tcga-39-5030 | dead | 59 | 59 | female | 81 | m0 | n2 | t2a | 3 |
|  | tcga-39-5029 | dead | 740 | NA | male | 67 | m0 | n2 | t1b | 3 |
|  | tcga-39-5028 | dead | 52 | 52 | male | 75 | m0 | n1 | t4 | 3 |
|  | tcga-39-5027 | alive | NA | 1849 | male | 73 | m0 | n0 | t2a | 1 |
|  | tcga-39-5024 | alive | NA | 2130 | female | 65 | m0 | n2 | t2a | 3 |
|  | tcga-39-5022 | dead | 1679 | NA | male | 76 | m0 | n0 | t2a | 1 |
|  | tcga-39-5021 | dead | 2086 | 2057 | male | 70 | m0 | n0 | t2a | 1 |
|  | tcga-39-5019 | alive | NA | 1361 | male | 70 | m0 | n0 | t2a | 1 |
|  | tcga-39-5016 | alive | NA | 2253 | male | 44 | m0 | n1 | t2a | 2 |
|  | tcga-39-5011 | alive | NA | 1454 | female | 70 | m0 | n0 | t1b | 1 |
|  | tcga-37-a5en | alive | NA | 660 | male | 59 | m0 | n2 | t4 | 3 |
|  | tcga-37-a5em | alive | NA | 867 | male | 49 | m0 | n0 | t2 | 2 |
|  | tcga-37-a5el | alive | 1143 | NA | male | 53 | m0 | n0 | t3 | 2 |
|  | tcga-37-5819 | alive | NA | NA | male | 64 | m0 | n2 | t2 | 3 |
|  | tcga-37-4141 | alive | NA | NA | female | 65 | m0 | n0 | t1b | 1 |
|  | tcga-37-4135 | alive | NA | NA | male | 68 | m0 | n0 | t2a | 1 |
|  | tcga-37-4133 | alive | NA | NA | male | 63 | m0 | n0 | t4 | 3 |
|  | tcga-37-4132 | alive | NA | 227 | female | 61 | m1 | n0 | t2 | 4 |
|  | tcga-37-4130 | alive | NA | NA | male | 56 | m0 | n0 | t1b | 1 |
|  | tcga-37-4129 | alive | NA | 242 | female | 52 | m0 | n0 | t1b | 1 |
|  | tcga-37-3792 | alive | NA | NA | male | 69 | m0 | n0 | t2 | 1 |
|  | tcga-37-3789 | alive | NA | NA | male | 65 | NA | n0 | t2 | 1 |
|  | tcga-37-3783 | alive | NA | NA | male | 51 | m0 | n2 | t3 | 3 |
|  | tcga-34-a5ix | alive | NA | 1031 | male | 80 | m0 | n0 | t3 | 2 |
|  | tcga-34-8456 | alive | NA | 804 | female | 71 | m0 | n1 | t2a | 2 |
|  | tcga-34-8455 | dead | 123 | NA | male | 67 | m1a | n0 | t4 | 4 |
|  | tcga-34-8454 | alive | NA | 827 | female | 62 | m0 | n1 | t3 | 3 |
|  | tcga-34-7107 | dead | NA | NA | male | 70 | m0 | n0 | t2a | 2 |
|  | tcga-34-5929 | dead | 151 | NA | female | 78 | m0 | n0 | t2 | 1 |
|  | tcga-34-5928 | alive | NA | 552 | female | 83 | m0 | n1 | t2 | 2 |
|  | tcga-34-5927 | alive | NA | 941 | female | 70 | m0 | n0 | t1 | 1 |
|  | tcga-34-5241 | alive | 515 | NA | male | 79 | m0 | n0 | t2 | 1 |
|  | tcga-34-5240 | alive | NA | 365 | female | 73 | m0 | n1 | t2 | 2 |
|  | tcga-34-5239 | alive | NA | 707 | male | 75 | m0 | n0 | t4 | 3 |
|  | tcga-34-5236 | dead | 276 | NA | male | 60 | m0 | n0 | t3 | 2 |
|  | tcga-34-5234 | alive | NA | 1715 | female | 71 | m0 | n0 | t1 | 1 |
|  | tcga-34-5232 | alive | NA | 2062 | female | 75 | m0 | n1 | t1 | 2 |
|  | tcga-34-5231 | alive | 1984 | NA | male | 72 | m0 | n0 | t1 | 1 |
|  | tcga-34-2608 | dead | 1000 | 1000 | male | 84 | m0 | n0 | t2 | 1 |
|  | tcga-34-2600 | dead | 1874 | NA | female | 76 | m0 | n0 | t1 | 1 |
|  | tcga-34-2596 | dead | 80 | 80 | male | 70 | m0 | n1 | t2 | 2 |
|  | tcga-33-aasl | dead | NA | NA | female | 57 | mx | n0 | t1 | 1 |
|  | tcga-33-aasj | dead | NA | NA | male | 60 | mx | n0 | t2 | 1 |
|  | tcga-33-aasi | dead | NA | NA | female | 65 | mx | n1 | t2 | 2 |
|  | tcga-33-aasd | dead | NA | NA | male | 83 | mx | n0 | t1 | 1 |
|  | tcga-33-aasb | dead | NA | NA | male | 66 | mx | n0 | t2 | 1 |
|  | tcga-33-aas8 | dead | NA | NA | female | 59 | mx | n0 | t1 | 1 |
|  | tcga-33-a5gw | alive | 9 | NA | male | 67 | mx | n1 | t1a | 2 |
|  | tcga-33-a4wn | dead | 143 | NA | male | 60 | mx | n0 | t2a | 1 |
|  | tcga-33-6738 | alive | NA | 822 | male | 80 | mx | n2 | t1 | 3 |
|  | tcga-33-6737 | dead | 601 | NA | male | 71 | m0 | n2 | t2 | 3 |
|  | tcga-33-4589 | dead | 47 | NA | female | 62 | m0 | n1 | t2 | 2 |
|  | tcga-33-4587 | dead | 1656 | NA | female | 63 | mx | n0 | t2 | 1 |
|  | tcga-33-4586 | dead | 428 | NA | male | 57 | m0 | n2 | t2 | 3 |
|  | tcga-33-4583 | alive | 4601 | NA | male | 73 | m0 | n0 | t1 | 1 |
|  | tcga-33-4582 | dead | 3149 | NA | male | 55 | m0 | n0 | t1 | 1 |
|  | tcga-33-4566 | dead | 5287 | NA | male | 40 | m0 | n0 | t2 | 1 |
|  | tcga-33-4547 | alive | NA | 2419 | male | 68 | m0 | n0 | t2 | 1 |
|  | tcga-33-4538 | dead | 2979 | NA | male | 66 | m0 | n2 | t2 | 3 |
|  | tcga-33-4533 | alive | NA | 4068 | female | 76 | m0 | n0 | t2 | 1 |
|  | tcga-33-4532 | alive | NA | 2524 | male | 68 | m0 | n0 | t2 | 1 |
|  | tcga-22-a5c4 | alive | NA | 671 | male | 70 | m0 | n0 | t2b | 2 |
|  | tcga-22-5492 | dead | 506 | 506 | female | 73 | m0 | n2 | t2a | 3 |
|  | tcga-22-5491 | dead | 1713 | 1713 | male | 74 | m0 | n0 | t1a | 1 |
|  | tcga-22-5489 | dead | 1912 | 1912 | male | 64 | m0 | n0 | t1b | 1 |
|  | tcga-22-5485 | dead | 916 | 916 | female | 58 | m0 | n0 | t1a | 1 |
|  | tcga-22-5483 | dead | 573 | 573 | male | 74 | m0 | n1 | t1a | 2 |
|  | tcga-22-5482 | dead | 357 | 357 | male | 81 | m0 | n0 | t2a | 1 |
|  | tcga-22-5481 | dead | 2409 | NA | female | 72 | m0 | n1 | t2 | 2 |
|  | tcga-22-5480 | dead | 2170 | 2170 | female | 66 | m0 | n0 | t1b | 1 |
|  | tcga-22-5479 | alive | NA | 1651 | male | 64 | m0 | n0 | t2 | 1 |
|  | tcga-22-5478 | dead | 24 | 24 | male | 79 | m0 | n0 | t2a | 1 |
|  | tcga-22-5477 | dead | 1346 | 1346 | male | 65 | m0 | n0 | t1 | 1 |
|  | tcga-22-5474 | dead | 445 | 445 | male | 74 | m0 | n0 | t2a | 1 |
|  | tcga-22-5473 | dead | 1933 | NA | male | 78 | m0 | n0 | t3 | 2 |
|  | tcga-22-5472 | dead | 1975 | 1975 | male | 67 | m0 | n0 | t2a | 1 |
|  | tcga-22-5471 | alive | NA | 965 | male | 75 | m0 | n0 | t2 | 1 |
|  | tcga-22-4613 | dead | 358 | 358 | female | 73 | m0 | n0 | t1b | 1 |
|  | tcga-22-4609 | dead | 291 | NA | male | 81 | m0 | n0 | t1 | 1 |
|  | tcga-22-4607 | dead | 587 | 587 | male | 75 | m0 | n0 | t2a | 1 |
|  | tcga-22-4605 | dead | 974 | NA | female | 78 | m0 | n0 | t2 | 1 |
|  | tcga-22-4604 | dead | 399 | NA | male | 73 | m0 | n1 | t2a | 2 |
|  | tcga-22-4601 | dead | 1057 | 1057 | female | 73 | m0 | n0 | t4 | 3 |
|  | tcga-22-4599 | dead | 1161 | 1161 | female | 73 | m0 | n0 | t2a | 1 |
|  | tcga-22-4596 | dead | 17 | 17 | female | 69 | m0 | n0 | t1b | 1 |
|  | tcga-22-4595 | dead | 734 | 734 | male | 57 | NA | n2 | t3 | 3 |
|  | tcga-22-4594 | dead | 1470 | 1470 | female | 60 | m0 | n2 | t3 | 3 |
|  | tcga-22-4593 | dead | 1067 | 1067 | male | 77 | m0 | n0 | t2b | 2 |
|  | tcga-22-4591 | dead | 623 | 623 | male | 80 | m0 | n2 | t3 | 3 |
|  | tcga-22-1017 | dead | NA | NA | male | 62 | m0 | n0 | t1 | 1 |
|  | tcga-22-1016 | dead | 822 | 802 | male | 65 | m0 | n0 | t2 | 1 |
|  | tcga-22-1012 | dead | 429 | NA | female | 80 | m0 | n0 | t2 | 1 |
|  | tcga-22-1011 | dead | 53 | 53 | male | 73 | m0 | n0 | t2 | 1 |
|  | tcga-22-1005 | dead | 1953 | 1953 | male | 63 | m0 | n0 | t1 | 1 |
|  | tcga-22-1002 | dead | 131 | 131 | male | 69 | m0 | n0 | t1 | 1 |
|  | tcga-22-1000 | dead | 454 | NA | male | 76 | m0 | n0 | t2 | 1 |
|  | tcga-22-0944 | dead | 223 | 223 | male | 61 | m0 | n0 | t2 | 1 |
|  | tcga-22-0940 | dead | 669 | 608 | male | 71 | m0 | n1 | t1 | 2 |
|  | tcga-21-a5di | alive | NA | 979 | male | 77 | m0 | n0 | t1b | 1 |
|  | tcga-21-5787 | dead | 329 | 329 | male | 65 | m0 | n2 | t2 | 3 |
|  | tcga-21-5786 | alive | NA | 661 | male | 64 | m0 | n0 | t2 | 1 |
|  | tcga-21-5784 | alive | NA | 908 | female | 80 | m0 | n0 | t2 | 1 |
|  | tcga-21-5783 | dead | 2680 | NA | male | 76 | m0 | n0 | t2 | 1 |
|  | tcga-21-5782 | dead | 962 | 962 | female | 68 | m0 | n0 | t2 | 1 |
|  | tcga-21-1083 | dead | 1315 | NA | male | 75 | m0 | n0 | t1 | 1 |
|  | tcga-21-1082 | alive | NA | 3644 | male | 61 | m0 | n0 | t2 | 1 |
|  | tcga-21-1081 | dead | 284 | 284 | male | 69 | m0 | n1 | t2 | 2 |
|  | tcga-21-1080 | alive | NA | 3724 | male | 66 | m0 | n0 | t2 | 1 |
|  | tcga-21-1079 | dead | 965 | 965 | male | 71 | m0 | n0 | t3 | 3 |
|  | tcga-21-1078 | dead | 474 | 469 | male | 77 | m0 | n0 | t2 | 1 |
|  | tcga-21-1077 | dead | 1058 | 1058 | male | 64 | m0 | n1 | t2 | 2 |
|  | tcga-21-1076 | alive | NA | 1852 | female | 54 | m0 | n0 | t2 | 1 |
|  | tcga-21-1075 | alive | NA | 2134 | male | 57 | m0 | n1 | t2 | 2 |
|  | tcga-21-1072 | alive | NA | 3016 | male | 75 | m0 | n0 | t2 | 1 |
|  | tcga-21-1071 | dead | 1426 | 1426 | male | 67 | m0 | n0 | t2 | 1 |
|  | tcga-21-1070 | alive | NA | 3636 | female | 60 | m0 | n0 | t3 | 3 |
|  | tcga-18-5595 | dead | NA | NA | male | 50 | m0 | n0 | t2 | 1 |
|  | tcga-18-5592 | alive | NA | 1519 | male | 57 | m0 | n0 | t3 | 2 |
|  | tcga-18-4721 | alive | NA | 4694 | male | 74 | m0 | n0 | t1 | 1 |
|  | tcga-18-4086 | dead | NA | NA | male | 64 | m0 | n0 | t2 | 1 |
|  | tcga-18-4083 | dead | 188 | NA | male | 63 | m0 | n1 | t2 | 2 |
|  | tcga-18-3421 | alive | NA | 2645 | male | 65 | m0 | n0 | t2 | 1 |
|  | tcga-18-3419 | alive | NA | 2811 | male | 73 | m0 | n1 | t2 | 2 |
|  | tcga-18-3417 | alive | 1097 | NA | male | 65 | m1 | n1 | t2 | 4 |
|  | tcga-18-3416 | alive | 973 | NA | male | 83 | m0 | n1 | t2 | 2 |
|  | tcga-18-3415 | alive | 2803 | NA | male | 77 | m0 | n0 | t2 | 1 |
|  | tcga-18-3414 | dead | 716 | NA | male | 73 | m1 | n1 | t4 | 4 |
|  | tcga-18-3412 | dead | 345 | NA | male | 52 | m0 | n0 | t2 | 1 |
|  | tcga-18-3411 | alive | NA | 3576 | female | 63 | m0 | n2 | t2 | 3 |
|  | tcga-18-3410 | dead | 146 | NA | male | 81 | m0 | n0 | t3 | 2 |
|  | tcga-18-3409 | alive | NA | 3747 | male | 74 | m0 | n0 | t1 | 1 |
|  | tcga-18-3408 | alive | 2304 | NA | female | 77 | m0 | n0 | t2 | 1 |
|  | tcga-18-3407 | dead | 136 | NA | male | 72 | m0 | n0 | t2 | 1 |
|  | tcga-18-3406 | dead | 371 | NA | male | 67 | m0 | n0 | t1 | 1 |
