# Supplementary material for: Four hub genes regulate tumor infiltration by immune cells, antitumor immunity in the tumor microenvironment, and survival outcomes in lung squamous cell carcinoma patients
Source: Aging (Albany NY). 2021 Jan 10;13(3):3819–42. doi: 10.18632/aging.202351 (PMC7906216; doi:10.18632/aging.202351)
Supplement: Supplementary File 2 [file aging-13-202351-s004.docx]

Supplementary File 2. The top 100 differentially upregulated genes in the TOX^WT^ CD8+ T cells compared to the TOX^∆^ CD8+ T cells in the GSE131643 dataset.

gene DAPL1 MAX DNAH8 COPG2 DDX6 KIF1B PLCB3 SYNE1 WDR82 CBL ZC3H12D DOCK6 IL6ST RNASEL CRIM1 PKN3 SSH2 XYLT1 SFMBT2 KAT6A PLD2 ARID5A BICRA SIK2 FAM120C MYO5A GPR137B LCA5 SNAP47 ARAP1 MAN2A1 SDCCAG8 VPS13B FAM129A BPHL IRAK2 CDK5RAP2 ERBB3 CARD11 PPP1R13B ABCC4 LNPEP ABTB2 HPS5 GAS7 SLC28A2 CHMP4B FBXO18 PIK3CD BDP1 ATM TMEM156 IKBKE CCDC91 NOTCH2 MPPE1 SLC26A2 ELOVL6 CPNE3 FAM214A AP5B1 SMARCA2 MYO1F SLC39A10 ANKZF1 WDR7 ATP10D BAZ2A IRF2BPL SCN4B GAS8 PRDM2 RABEP2 FAM13B SORL1 PRDM16 PPP1R21 CLASP2 N4BP1 RABGAP1 HOOK3 RNF215 TRIM14 IQGAP1 FCHSD2 DNAJC16 SLC26A6 MAST4 FLOT1 CLIP1 DHX29 NBR1 NAGPA EVPL ANKRD52 PIK3R5 DHX34 CFAP43 PDE4DIP SLC2A1
